# A knock-in mouse model for *GABRG2*-related epileptic encephalopathy displays spontaneous generalized seizures and cognitive impairment

**DOI:** 10.1038/s41420-025-02759-4

**Published:** 2025-10-06

**Authors:** Dingding Shen, Jiali Wan, Xin Zhang, Jiahui Sui, Longwu Zhan, Yuqin Zheng, Yaohui Ni, Qi Zhang

**Affiliations:** 1 Department of Neurology in Affiliated Hospital of Nantong University, Key Laboratory of Neuroregeneration of Jiangsu and Ministry of Education, Medical School, Co-innovation Center of Neuroregeneration, Nantong University, Nantong, China; 2 Key Laboratory of Neuroregeneration of Jiangsu and Ministry of Education, Co-innovation Center of Neuroregeneration, NMPA Key Laboratory for Research and Evaluation of Tissue Engineering Technology Products, Nantong University, Nantong, China; 3 Department of Pediatrics, Affiliated Hospital of Nantong University, Medical School, Nantong University, Nantong, China; 4 The Key Laboratory of Children’s Disease Research in Guangxi’s Colleges and Universities, Education Department of Guangxi Zhuang Autonomous Region, Nanning, China

**Keywords:** Epilepsy, Cell death in the nervous system

## Abstract

De novo mutations in voltage- and ligand-gated ion channels have been associated with an increasing number of cases of developmental and epileptic encephalopathies (DEEs), which often fail to respond to classic antiseizure medications. A de novo mutation (c.C316G > A, p.A106T) in the human GABA type-A receptor γ2 subunit gene (*GABRG2*) has been recurrently identified in patients with DEE. In this study, we generated a knock-in mouse model replicating the human *GABRG2(A106T)* variation (*Gabrg2*^*+/A105T*^ in mouse). *Gabrg2*^*+/A105T*^ mice displayed early mortality, spontaneous seizures, and heightened seizure susceptibility. Behavioral analysis revealed phenotypes consistent with DEE, including impaired spatial learning and memory, as well as increased anxiety-like behavior. Reduced γ2 subunit protein expression was detected in the hippocampus of mutant mice, but not other brain regions. Electrophysiological recordings revealed a significant decrease in the amplitude of miniature inhibitory postsynaptic currents (mIPSCs), indicating impaired synaptic GABAergic inhibition. Notably, hippocampal transcriptome profiling provided evidence of neuroinflammation, and histological analysis demonstrated neuronal loss and microglia activation prior to seizure onset. These findings indicate that neuroinflammatory processes, a major theme in acquired epilepsies, may potentially exacerbate epileptogenesis in *Gabrg2*^*+/A105T*^ mice. The knock-in mouse model serves as a potential model for evaluating anti-inflammatory therapies as adjunct treatments for drug-resistant DEEs.

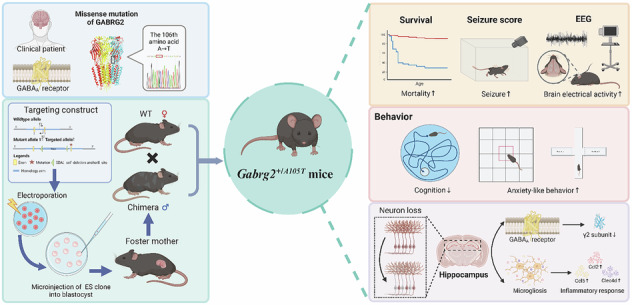

## Introduction

Developmental and epileptic encephalopathies (DEEs) comprise a large, heterogeneous group of severe neurodevelopmental disorders characterized by early-onset, severe seizures and encephalopathy, which involves significant developmental delay and cognitive impairment [1]. Current treatments for DEEs have limited efficacy in alleviating seizures and comorbidities, posing an urgent need to understand the etiology of DEEs to find new therapeutic targets [2]. The causes of DEEs are heterogeneous, but most commonly genetic [3]. Recent advances in genetic testing technologies have revolutionized the identification of a diverse array of genetic abnormalities in DEEs [4]. Recent discoveries of epilepsy-related genes in multiple laboratories and through the large Epi4K, EpiPM, and EuroEPINOMICS-RES consortia have identified a diverse array of proteins that may contribute to epileptogenesis [5–7]. Early diagnosis and treatment are essential to aid the reduction of the severity of symptoms and enable the best possible outcome, even if efforts are still needed to establish phenotype-genotype correlations. Among them, dominant variants have been found in the *GABRG2* gene, which encodes the γ2 subunits of ligand-gated γ-Aminobutyric acid type A (GABA_A_) receptors.

GABA_A_ receptors are a class of the Cys-loop ligand-gated ion channels and comprise five subunits arranged in a pseudosymmetrical arrangement. The archetypical GABA_A_ receptor subtypes are mostly heterotetrametric channels made up of two α subunits, two β subunits and a single γ or δ subunit [8]. GABA_A_ channels mediate inhibitory neurotransmission and are key for maintaining the excitatory/inhibitory balance throughout the brain. To date, hundreds of genetic variants in different GABA_A_ receptor subunit genes have been clearly linked to epilepsy, making them a prominent cause of genetically linked epilepsies [9, 10]. Expression of γ2 subunits is widespread and abundant in the embryonic and neonatal brain, especially in the cerebral cortex, hippocampus, and other regions involved in generating seizures [11]. The γ2 subunit plays an important role during brain development and has a critical role in GABA_A_ receptor trafficking and clustering at synapses. The phenotypic spectrum described for individuals with variants in the *GABRG2* gene ranged from mild forms within genetic epilepsy with febrile seizures plus (GEFS+) spectrum to severe forms of DEEs [12]. Notably, the mechanisms underlying this phenotypic diversity and risks of developing severe comorbidities associated with *GABRG2* are unclear [10].

While GABR variants were initially thought to cause epilepsy through loss-of-function (LOF) effects via impaired receptor expression and/or activation, this LOF-centric view fails to fully explain for the clinical heterogeneity [13]. A growing number of de novo or rare missense variants across several GABA_A_ receptor subunit genes-including *GABRA1*, *GABRA4*, *GABRB2*, *GABRB3*, and *GABRD* confer gain-of-function (GOF) effects-typically by increasing GABA potency or prolonging receptor activation [14–18]. Notably, GOF variants typically correlate with more severe clinical presentations than LOF variants [15]. Whether and how these functional changes—whether LOF or GOF—ultimately drive epileptic seizures and behavioral deficits in DEEs remain to be fully elucidated.

Among these variants, a mutation of alanine at amino acid position 106 to threonine (A106T) was originally identified by our team in a prior study [19] and has since been recurrently reported in patients with DEE. This mutation was found in two patients who displayed drug-resistant neonatal tonic-clonic seizures and later developed profound intellectual and language disability, spasticity, and autistic behavior [19]. Located in the β1-β2 loop of the N-terminal domain, the *GABRG2(A106T)* mutation led to LOF in heterologous cells, characterized by reduced surface expression of heteromeric channels, decreased GABA potency, slowed desensitization, accelerated receptor activation and slowed deactivation of GABA_A_ receptor [19]. While in vitro functional study combined with the genetic information suggests that *GABRG2(A106T)* mutation may be major contributor to the epilepsy phenotypes, a causal role of this mutation can only be established directly from developing a mouse model harboring this mutation. In this study, we investigated the contribution of the DEE variant *GABRG2(A106T)* by generating and characterizing a knock-in (KI) mouse model, the heterozygous *Gabrg2*^*+/A105T*^ mouse, corresponding to the heterozygous *GABRG2(A106T)* mutation in humans. A106 in the human GABRG2 is conserved in the mouse GABRG2 at amino acid position 105. These mice displayed spontaneous seizures, cognitive deficits, and widespread neurodegeneration with reactive microgliosis in the hippocampus, providing a causal link between A105T-mediated disruption of GABA_A_ receptor function and *GABRG2*-associated DEE.

## Results

### Generation of *Gabrg2*^*+/A105T*^ mice

**Fig. 1:**
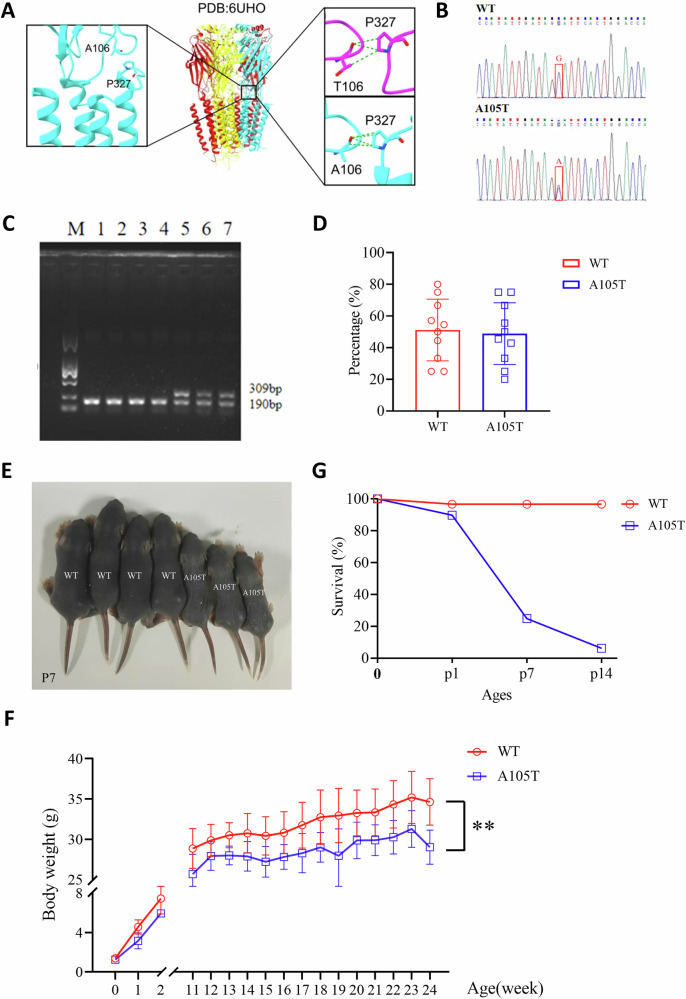
Phenotypic characterization of the *Gabrg2*^*+/A105T*^ knock-in mouse model. **A** Structural modeling of the human full-length heteromeric α1β3γ2L GABA_A_R. Left panel: interaction analysis of γ2A106 with residues within the γ2 subunit (γ2L192 and γ2P327). Middle panel: structural model of the WT GABA_A_R complex. Right panel: predicted structural changes in the γ2T106 variant, showing weakened interactions between γ2T106 and γ2P327 near the transmembrane domain. **B** Genotyping results of *Gabrg2*^*+/A105T*^ mice showing the mutation of “G” to “A”. **C** Representative gel image of genotyping PCR products. Heterozygous *Gabrg2*^*+/A105T*^ mice show two bands (309 bp and 190 bp), while wild-type (WT) mice show a single band (190 bp). **D** The percentage of WT and heterozygous *Gabrg2*^*+/A105T*^ mice after birth (n = 10). **E** Gross morphology of *Gabrg2*^*+/A105T*^ and WT mice at postnatal day 7 (P7). **F** Body weight analysis of *Gabrg2*^*+/A105T*^ and WT mice from birth to adulthood (n = 9–10). **G** Survival plot showing increased early mortality in *Gabrg2*^*+/A105T*^ mice compared to WT littermates. Approximately 75.1% of *Gabrg2*^*+/A105T*^ mice died before 1 week of age, with a peak mortality around postnatal day 7. Data are presented as mean ± SD and ** *P* < 0.01 using two-way ANOVA followed by Sidak’s multiple comparisons test.

To understand the structural impact of the γ2(A106T) variant, we performed structural modeling based on the human full-length heteromeric α1β3γ2L GABA_A_R (Fig. 1A, middle panel). Dynamut calculations revealed a ΔΔGStability of −1.2 kcal/mol for γ2T106, indicating that the A-to-T mutation negatively affects the overall stability of the complex. Further structural analysis (Fig. 1A, left panel) showed that γ2A106 interacts exclusively with other residues within the γ2 subunit (γ2L192 and γ2P327) without interacting with other subunits. Subsequently, we used Chai-1 to further predict the structure of A106T and compared it with the wild-type GABA_A_R. The results showed that the interaction between γ2T106 and γ2P327, located near the transmembrane domain, was weaker than that in the γ2A106 wild type (Fig. 1A, right panel). This weakened interaction may reduce the stability of the transmembrane region of the γ2 subunit, potentially affecting the membrane localization of the entire complex.

The mouse model was generated by homologous recombination in embryonic stem cells using a targeting vector containing regions homologous to the genomic *Gabrg2* sequences and the p.(A105T) variant, which corresponds to A106T in the long isoform of human GABRG2 subunit. DNA sequencing showed a mutagenesis of the *GABRG2* coding sequence led to the c.G313A, p. A105T mutation (Fig. 1B) and the genotype was confirmed by PCR (Fig. 1C). The *Gabrg2*^*+/A105T*^ mice were maintained on C57BL/6 genetic background. Mating of male KI mice with female WT mice revealed a ratio of KI to WT offspring at weaning that was consistent with Mendel’s law (Fig. 1D).

*Gabrg2*^*+/A105T*^ mice do not display gross morphological abnormalities but have reduced weight compared to WT littermates (Fig. 1E, F). Although both the mutant pups and the WT pups exhibited comparable weights at birth, growth impairment began after birth and exacerbated throughout infancy and juvenile period into adulthood (*P* < 0.05 from 11 weeks, Fig. 1F). Dissected brains from 10-to 12-week-old *Gabrg2*^*+/A105T*^ mice appeared similar in morphology and size to their WT littermates, suggesting that expression of the γ2(A105T) subunits does not grossly alter brain development. At necropsy, no significant macroscopic organ abnormalities and histological alterations were observed in heart, liver, spleen, lungs, or kidneys (Supplementary Fig. 1).

### *Gabrg2*^*+/A105T*^ pre-weaned mice had premature sudden death and increased mortality

Sudden Unexplained Death in Epilepsy (SUDEP) is commonly observed in patients with DEE. We first observed that many pups died in their home cage prior to weaning. In the first two-week postnatal period, KI pups died at a much higher rate (93.8% died vs 6.2% survived) than WT mice (3.3% died vs 96.7% survived), consistent with SUDEP in humans. Systematic home cage video monitoring showed that the dead mice had frequent generalized tonic-clonic seizures (GTCS) before their death. A significant proportion of *Gabrg2*^*+/A105T*^ mice (approximately 75.1%) die before 1 week of age, with a peak of mortality evidenced around postnatal day 7. Kaplan–Meir survival plot confirms increased early mortality in the *Gabrg2*^*+/A105T*^ mice (Fig. 1G).

### *Gabrg2*^*+/A105T*^ mice displayed spontaneous tonic-clonic seizures and increased sensitivity to PTZ

**Fig. 2:**
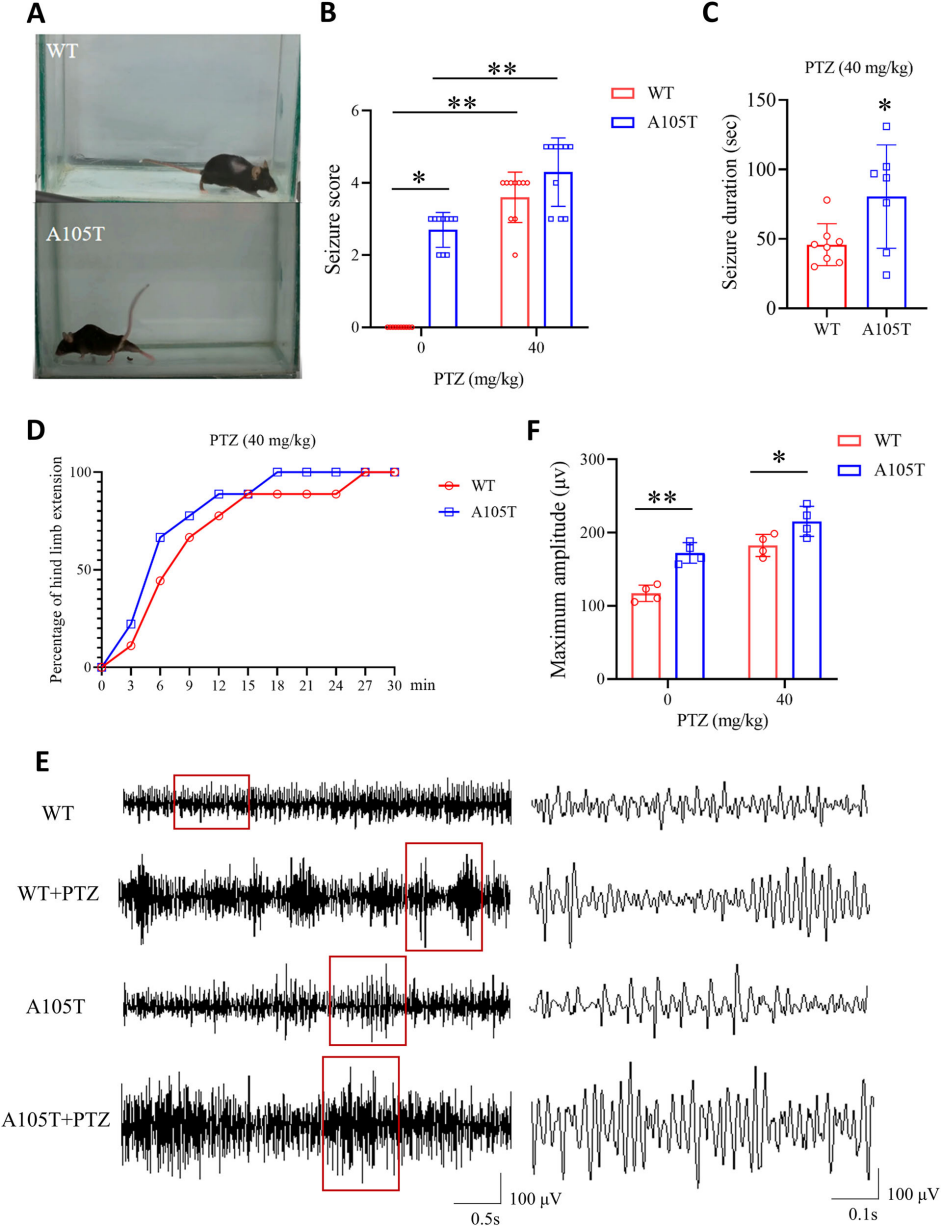
Spontaneous seizures and increased seizure susceptibility in *Gabrg2*^*+/A105T*^ mice. **A** Representative image of a *Gabrg2*^*+/A105T*^ mouse exhibiting a rigid posture and extended legs during a spontaneous convulsive seizure. **B** Seizure scores in *Gabrg2*^*+/A105T*^ mice compared to WT littermates following intraperitoneal injection of PTZ (40 mg/kg) (n = 10 per group). **C** Seizure duration in *Gabrg2*^*+/A105T*^ and WT mice induced with PTZ injection (n = 8 for WT and n = 7 for A105T). **D** Latency to hind limb extension and percentage of mice reaching hind limb extension after PTZ injection. **E** Representative EEG recording from *Gabrg2*^*+/A105T*^ and WT mice with or without PTZ injection. **F** Maximal EEG amplitude in *Gabrg2*^*+/A105T*^ and WT mice (n = 4 per group). Data are presented as mean ± SD and * *P* < 0.05, ** *P* < 0.01 using Kruskal–Wallis test (2B), Unpaired t-test (2C) or two-way ANOVA followed by Sidak’s multiple comparisons test (2F).

The sudden death phenotype observed in *Gabrg2*^*+/A105T*^ mice, along with the epilepsy syndromes seen in patients carrying the *GABRG2* p.A106T variant, suggests the animals may be undergoing GTCS, which in mice often result in death when escalating into tonic hindlimb extension. One such death event was indeed observed in a male KI mouse, and several convulsive seizure events were witnessed during routine cage inspections. One *Gabrg2*^*+/A105T*^ mouse with a rigid posture and extended legs was presented in Fig. 2A and Supplementary Video 1. *Gabrg2*^*+/A105T*^ mice aged 4 to 6 months had increased seizure scores compared to WT littermates (*P* < 0.01, Fig. 2B).

To examine whether mutant γ2(A105T) subunits affect seizure susceptibility, we compared PTZ-induced seizure threshold for *Gabrg2*^*+/A105T*^ KI mice to their WT littermates. PTZ is a noncompetitive GABA_A_ receptor antagonist that is used widely to provoke seizures as a method to assess central nervous system network excitability and seizure susceptibility. KI mice and WT littermates (between 4 and 6 months old) were injected intraperitoneally with PTZ, and the seizure duration and latencies to the onset of GTCS, characterized by hind limb extension were recorded. At a dose of 40 mg/kg, KI mice had increased seizure duration compared to WT littermates (*P* < 0.05, Fig. 2C). The number of animals reach hind limb extension doubled in *Gabrg2*^*+/A105T*^ mice compared to the WT littermates 3 min after PTZ injection (Fig. 2D). Seizure onset was induced in all the *Gabrg2*^*+/A105T*^ mice 18 min after PTZ injection while the duration extended to 27 min in the WT control (Fig. 2D). These results indicated that mutant γ2 (A105T) subunits affected the brain’s neuronal inhibition level and significantly lowered the PTZ-induced seizure threshold.

To further observe the seizure onset, EEG recordings were performed for 4 heterozygous animals (2 males and 2 females) and compared to 4 WT littermates (2 males, 2 females). Each animal was recorded, and the observed paroxysmal events were performed. The recording of one of those seizures from a *Gabrg2*^*+/A105T*^ mouse is represented in Fig. 2E. The maximal amplitude observed in the *Gabrg2*^*+/A105T*^ mice was significantly higher than that in the WT controls, both with (*P* < 0.05) and without (*P* < 0.01) PTZ induction (Fig. 2F).

### *Gabrg2*^*+/A105T*^ mice had impaired spatial learning and memory, and elevated anxiety

**Fig. 3:**
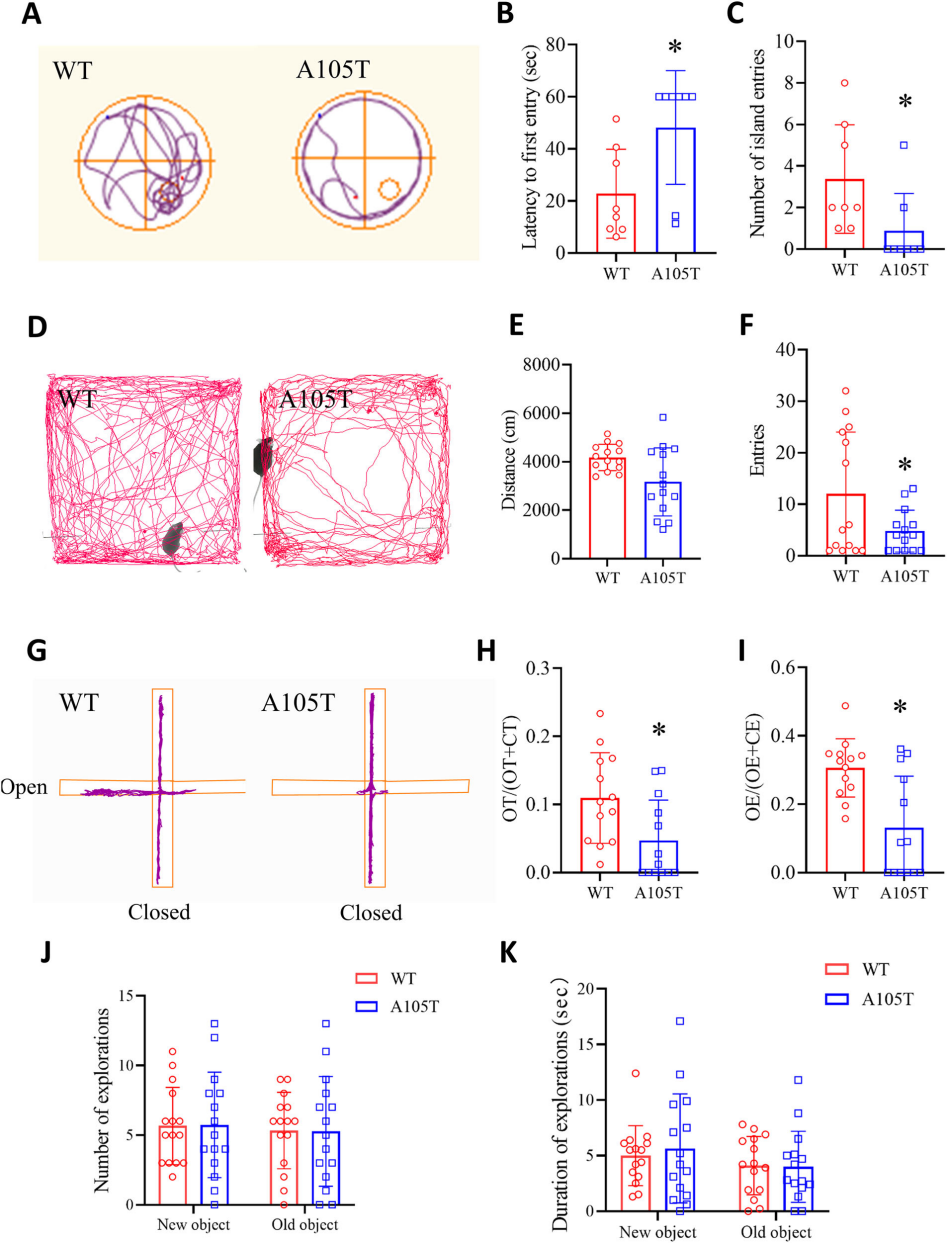
Behavioral deficits in *Gabrg2*^*+/A105T*^ mice. **A** Swimming speed and thigmotaxis in the Morris water maze test. **B** Escape latency in the Morris water maze *Gabrg2*^*+/A105T*^ (n = 8 per group). **C** Number of entries into the platform quadrant in the Morris water maze (n = 8 per group). **D** Movement trajectory diagram of mice during open field test. **E** Total distance traveled in the open field test (n = 14 per group). **F** Number of entries into the central area of the open field (n = 14 per group). **G** Schematic of the Elevated Plus Maze (EPM) apparatus used to assess anxiety-like behavior. **H** Percentage of time spent in the open arms of the EPM (n = 13 per group). **I** Number of entries into the open arms of the EPM (n = 13 per group). **J** Exploration time of novel and familiar objects in the Novel Object Recognition test (n = 15 per group). **K** Frequency of exploration of novel and familiar objects (n = 15 per group). Data are presented as mean ± SD and * *P* < 0.05 vs. WT using Unpaired Student’s *t* test (**B**, **C**, **F**, **H**, **I**), Mann–Whitney U test (**E**) or two-way ANOVA followed by Sidak’s multiple comparisons test (**J**, **K**).

Patients with the *GABRG2(A106T)* variant have been reported to experience profound intellectual disability and neuropsychiatric comorbidities along with seizures. To examine the effect of *GABRG2(A105T)* on these behavioral alterations, we subjected KI mice and WT littermates aged between 4 and 6 months to a battery of neurobehavioral tests (Fig. 3).

The performance of mutant mice in hippocampus-dependent spatial learning and memory tests may be affected by altered excitability of hippocampal neurons. We exploited the Morris water maze to test hippocampus-dependent learning and memory performance in mutants (Fig. 3A). In this test, during the acquisition phase, rodents had to learn the position of a submerged resting platform beneath opaque water using spatial cues. The mutants showed normal thigmotaxis (swimming close to the pool wall) compared to controls. However, *Gabrg2*^*+/A105T*^ mice showed an increased escape latency and decreased entries to the platform quadrant compared to control mice (*P* < 0.05, Fig. 3B, C). The swimming velocity and distance of *Gabrg2*^*+/A105T*^ mice were comparable to those of control mice (Supplementary Fig. 2A, B). To address the concern regarding visual function, we conducted Electroretinography (ERG) recordings and quantified flash visual evoked potential (VEP) amplitudes in mutant and WT mice. No significant genotype differences were detected (*P* > 0.05), indicating that the KI mice have intact visual ability (Supplementary Fig. 3).

In the open field test, the distance of *Gabrg2*^*+/A105T*^ mice traveled in the entire arena did not show a significant difference when compared to that of the WT control (*P* > 0.05, Fig. 3D, E). In the arena, anxious mice usually spend more time in the periphery (home) compared to the other regions in which the arena was divided (transition, exploration). *Gabrg2*^*+/A105T*^ mice appeared more anxious compared to control mice as they had less entries to the central area compared to the control group (*P* < 0.05, Fig. 3F). No difference in repetitive behaviors, including body rotations and grooming, occurring during the open field, was observed.

Then we examined the behavioral thigmotaxis and explorative response of mutant mice in the Elevated Plus Maze (EPM) apparatus, commonly used to evaluate anxiety response to a novel environment in rodents. The mice were positioned on the central platform with their tail pointed toward the closed arm nearest to the experimenter. Rodents were left free to explore the EPM for 5 min while a video camera placed orthogonally to the maze recorded from above (Fig. 3G). The distance traveled, the velocity, the number of closed arm entries, the closed arm stay time, the open arm entries, and the open arm stay time were computed by an automated system. As far the locomotor activity is concerned, *Gabrg2*^*+/A105T*^ mice and WT littermates did not differ for travel distance or velocity in the open arms (Supplementary Fig. 2C, D). *Gabrg2*^*+/A105T*^ mice spent less percent of time and less entries into open arms compared with WT littermates (*P* < 0.05, Fig. 3H, I). This rodent behavior has been interpreted as increased anxiety [20].

Finally, we applied New Object Recognition to determine the novelty recognition of the mutant mice. No significant differences in exploration time or frequency between novel and familiar objects were detected between the *Gabrg2*^*+/A105T*^ mice and WT littermates (*P* > 0.05, Fig. 3J, K). Taken together, the behavioral tests indicated that *Gabrg2*^*+/A105T*^ mice had impaired spatial learning and memory, and elevated anxiety.

### *Gabrg2*^*+/A105T*^ mice had decreased γ2 expression and impaired inhibitory synaptic transmission in hippocampal neurons

**Fig. 4:**
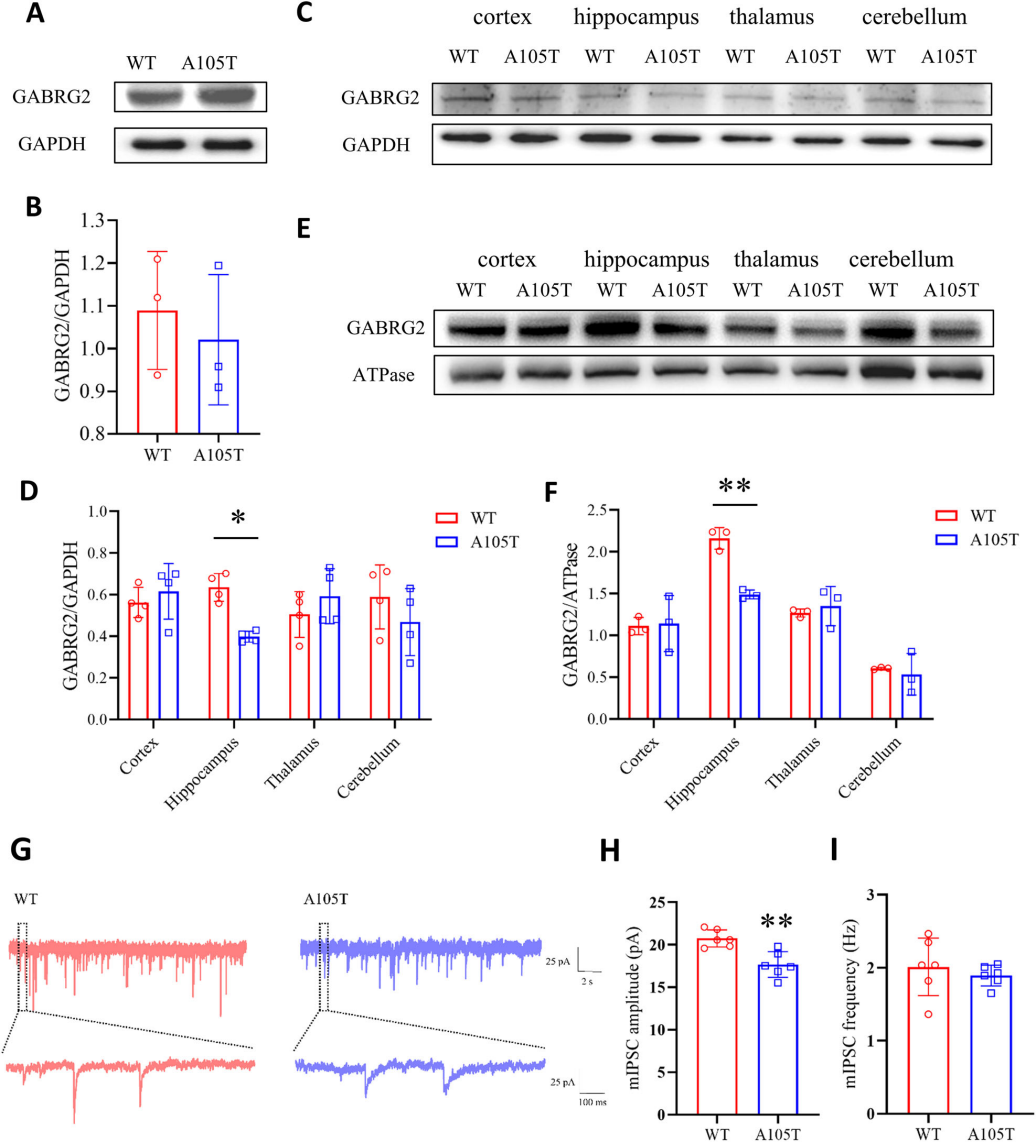
Altered γ2 subunit expression and hippocampal pathology in *Gabrg2*^*+/A105T*^ mice. **A** Representative Western blot images of γ2 subunit expression in the whole brain of *Gabrg2*^*+/A105T*^ and WT mice. **B** Quantification of total γ2 subunit expression in the whole brain of *Gabrg2*^*+/A105T*^ mice compared to WT controls (n = 3 per group). **C** Representative Western blot images of γ2 subunit expression in specific brain regions (cerebellum, cortex, hippocampus, and thalamus) of *Gabrg2*^*+/A105T*^ and WT mice. **D** Quantification of total γ2 subunit expression in specific brain regions (cerebellum, cortex, hippocampus, and thalamus) (n = 4 per group). **E** Representative Western blot images of membrane γ2 subunit expression in specific brain regions (cerebellum, cortex, hippocampus, and thalamus) of *Gabrg2*^*+/A105T*^ and WT mice. **F** Quantification of membrane γ2 subunit expression in specific brain regions (cerebellum, cortex, hippocampus, and thalamus) (n = 3 per group). **G** Example mIPSCs recorded from WT (left, red) and *Gabrg2*^*+/A105T*^ (right, blue) hippocampal neurons. **H** Average amplitudes of mIPSCs (n = 6 per group). **I** Average frequency of mIPSCs (n = 6 per group). Data are presented as mean ± SD and * *P* < 0.05, ** *P* < 0.01 vs. WT using two-way ANOVA followed by Sidak’s multiple comparisons test (**D**, **F**) or Unpaired t-test (**B**, **H**, **I**).

Having confirmed that KI mice had spontaneous seizures and behavioral phenotypes of DEE, we next explored the underlying mechanisms. Most GABR epilepsy mutations produce subunits with impaired biogenesis, leading to altered cell surface expression. To determine whether the A105T mutation affects γ2 subunit expression in vivo, we first quantified total protein levels in the whole brain, cerebellum, cortex, hippocampus, and thalamus. The *GABRG2(A105T*) mutation did not affect γ2 expression in the whole brain (Fig. 4A, B). Notably, we saw a significant decrease of γ2 subunits only in the hippocampus (*P* < 0.05, Fig. 4C, D). Next, we assessed the expression of GABA_A_ receptor subunits at membrane levels. Similarly, we saw a significant decrease of membrane γ2 subunits in the hippocampus (*P* < 0.01, Fig. 4E, F).

We next investigated whether mutant γ2(A105T) subunits impair inhibitory synaptic function in CA1 pyramidal neurons. Whole-cell patch-clamp recordings in acute coronal hippocampal slices from *Gabrg2*^*+/A105T*^ mice revealed a significant reduction in the amplitude of GABA_A_ receptor-mediated mIPSCs (*P* < 0.01; Fig. 4G, H), whereas mIPSC frequency remained unchanged (*P* > 0.05; Fig. 4I). The reduction in mIPSC amplitude suggests decreased postsynaptic responsiveness of GABA_A_ receptors containing the mutant γ2(A105T) subunit, potentially weakening phasic GABAergic inhibition and increasing neuronal hyperexcitability. These findings support a loss-of-function effect of the mutation on synaptic GABA_A_ receptor–mediated transmission and provide functional evidence of impaired inhibitory signaling underlying the observed behavioral and electrographic phenotypes.

### *Gabrg2*^*+/A105T*^ mice had neuronal loss in the hippocampus

**Fig. 5:**
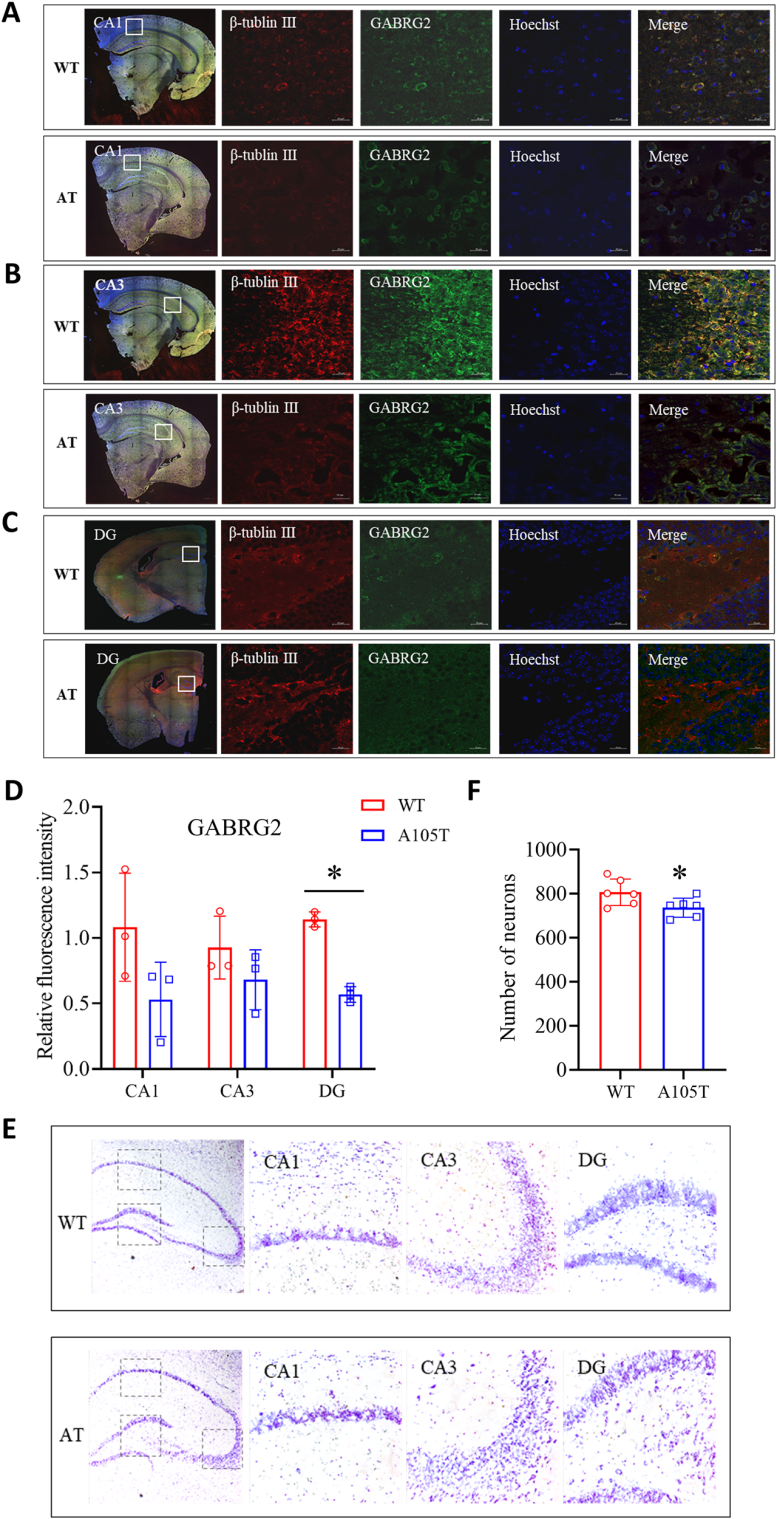
Immunofluorescence analysis of GABRG2 expression and localization in *Gabrg2*^*+/A105T*^ mice. Immunofluorescence staining of GABRG2 (green) and β-tubulin III (red) in the hippocampal CA1 (**A**), CA3 (**B**), and DG (**C**) regions of *Gabrg2*^*+/A105T*^ and WT mice. GABRG2 was predominantly localized on the neuronal membrane, with some cytoplasmic expression. Scale bars = 500 µm for the first column and = 20 µm for the rest. **D** Average fluorescence intensity of GABRG2 in the hippocampal CA1, CA3, and DG regions of *Gabrg2*^*+/A105T*^ and WT mice (n = 3 per group). **E** Nissl staining of hippocampal regions in *Gabrg2*^*+/A105T*^ and WT mice. Scale bars = 500 µm. **F** Quantification of number of neurons in the hippocampus. Data are presented as mean ± SD and * *P* < 0.05 vs. WT using two-way ANOVA followed by Sidak’s multiple comparisons test (5D) or Unpaired t-test (5F).

To further investigate the expression and localization of GABRG2 in mouse brain tissues, we performed immunofluorescence staining of the γ2 subunit in the hippocampal CA1 (Fig. 5A), CA3 (Fig. 5B), and DG (Fig. 5C) regions. Neurons were labeled with β-tubulin III (red), and GABRG2-positive cells were visualized in green. In both WT and A105T mutant mice, GABRG2 was predominantly localized to the neuronal membrane, with partial cytoplasmic expression. Although the spatial pattern of GABRG2-positive cells was comparable between genotypes, fluorescence intensity was markedly reduced in *Gabrg2*^*+/A105T*^ mice across all subregions, with the most pronounced reduction in the DG (*P* < 0.05; Fig. 5D). These findings suggest that the A105T mutation may impair the expression or stability of the γ2 subunit without affecting its overall subcellular localization.

Nissl staining revealed a modest but statistically significant reduction in neuronal density in the hippocampus of *Gabrg2*^*+/A105T*^ mice compared to WT controls (*P* < 0.05; Fig. 5E, F). While the overall magnitude of neuronal loss is limited, it may reflect early neurodegenerative vulnerability associated with γ2 subunit dysfunction.

### Altered transcriptome in the hippocampus of *Gabrg2*^*+/A105T*^ mice

**Fig. 6:**
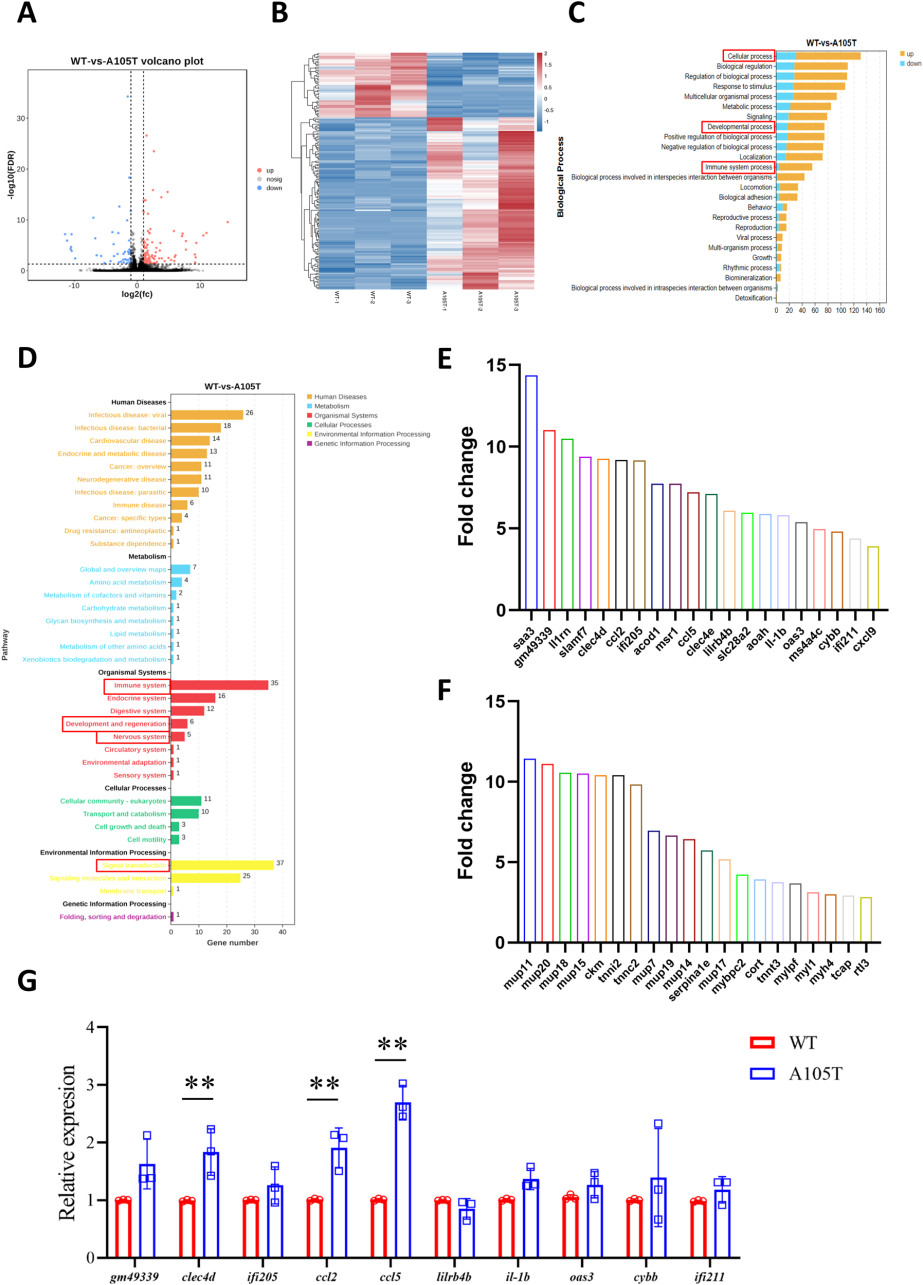
Transcriptomic analysis of hippocampal tissues from *Gabrg2*^*+/A105T*^ mice. **A** Volcano plot showing differentially expressed genes (DEGs) in the hippocampus of *Gabrg2*^*+/A105T*^ mice compared to WT controls. A total of 154 DEGs were identified (adjusted *P* < 0.05), including 112 up-regulated and 42 down-regulated genes. **B** Heatmap of the 154 DEGs, illustrating distinct gene expression patterns between *Gabrg2*^*+/A105T*^ and WT mice. **C** GO analysis of DEGs. Up-regulated and down-regulated genes were significantly enriched in cellular processes, developmental processes, and immune system processes. **D** KEGG pathway analysis of DEGs. Key pathways included signal transduction, immune system, development and regeneration, and nervous system functions. **E** Top 20 most significantly up-regulated genes in the hippocampus of *Gabrg2*^*+/A105T*^ mice. **F** Top 20 most significantly down-regulated genes in the hippocampus of *Gabrg2*^*+/A105T*^ mice. **G** qRT-PCR validation of immune-related genes in the hippocampus (n = 3 per group). Data are presented as mean ± SD and **P < 0.01 vs. WT using two-way ANOVA followed by Sidak’s multiple comparisons test.

To gain a deeper understanding of the molecular mechanisms underlying the epilepsy and behavioral comorbidities, we performed RNA-seq analysis using the hippocampal tissues from the WT and *Gabrg2*^*+/A105T*^ mice of 2-month-old (n = 3 mice per group). We focused on the hippocampus for two reasons: (1) it is the predominant region of seizure origin and maintenance. (2) *Gabrg2*^*+/A105T*^ mice had decreased γ2 expression and neuronal loss only in the hippocampus. Principal component analysis (PCA) revealed the global transcriptome variation across individual samples, supported by pairwise Pearson correlation coefficients (r) illustrating inter-sample relationships (Supplementary Fig. 4). We identified 154 differentially expressed genes (DEGs, adjusted *P* < 0.05, 112 up and 42 down) in the *Gabrg2*^*+/A105T*^ mice, compared to the control group (Fig. 6A, B).

GO analysis indicated that 100 up-regulated and 31 down-regulated genes were involved in cellular processes, while 57 up-regulated and 18 down-regulated genes were associated with developmental processes. Additionally, 52 up-regulated and 4 down-regulated genes were implicated in immune system processes (Fig. 6C). Further KEGG pathway analysis of the DEGs identified 37 genes related to signal transduction, 35 genes associated with the immune system, 6 genes involved in development and regeneration, and 5 genes linked to the nervous system (Fig. 6D). These findings suggested that the *GABRG2(A105T)* mutation significantly altered gene expression profiles in the hippocampus, potentially impacting cellular, developmental, and immune processes, as well as signal transduction and nervous system functions.

Based on the transcriptome sequencing results, we identified the top 20 most significantly up-regulated (Fig. 6E) and down-regulated (Fig. 6F) genes in the hippocampal tissues of *Gabrg2*^*+/A105T*^ mice compared to WT controls. Notably, among the up-regulated genes, several were associated with immune regulation. To validate these findings, we performed qRT-PCR analysis, which confirmed that the expression levels of immune-related genes, including *ccl5*, *ccl2*, and *clec4d*, were significantly elevated in the hippocampal tissues of *Gabrg2*^*+/A105T*^ mice compared to WT controls (*P* < 0.01, Fig. 6G). These results suggested that the *GABRG2(A105T)* mutation might dysregulate immune responses in the hippocampal tissue, potentially influencing hippocampal neuronal development. This immune dysregulation could contribute to the observed epileptic seizures and neurobehavioral alterations in *Gabrg2*^*+/A105T*^ mice. However, the precise mechanisms underlying these effects require further investigation.

In the PCA analysis of RNA-seq data, the A105T samples clustered distinctly from the control groups, but showed greater intra-group variability. While this variability does not undermine our core findings, it suggests that the A105T-induced transcriptomic signature may be inherently heterogeneous. Future studies with larger cohorts will be needed to better capture the full spectrum of this response.

### *Gabrg2*^*+/A105T*^ mice had neuroinflammation in the hippocampus before seizures

**Fig. 7:**
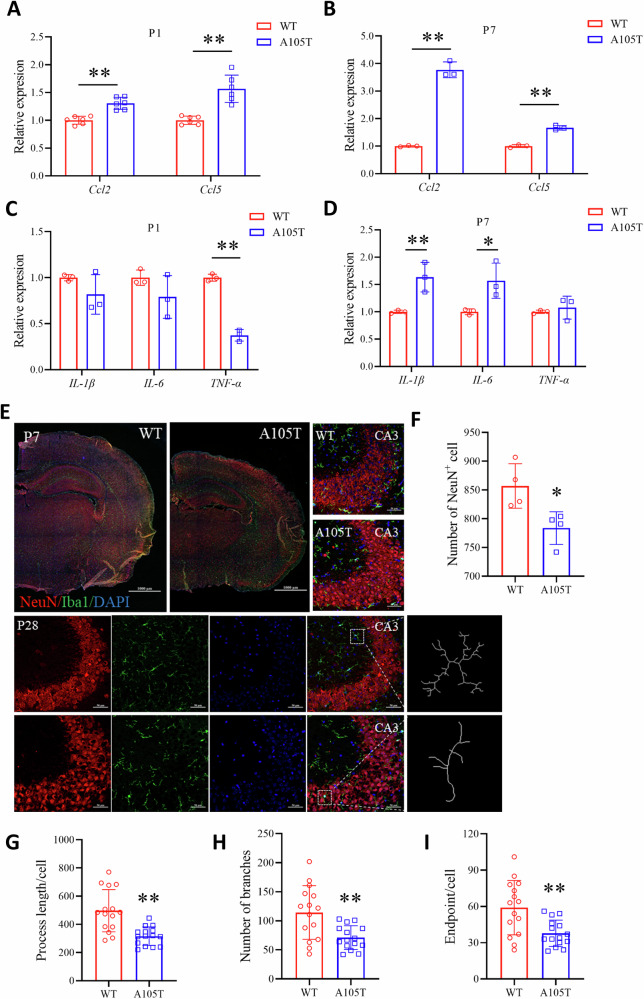
Neuroinflammatory changes and microglial activation in *Gabrg2*^*+/A105T*^ mice. **A**, **B** Expression levels of inflammatory factors *Ccl2* and *Ccl5* in the hippocampus of *Gabrg2*^*+/A105T*^ and WT mice at postnatal day 1 (P1) (n = 6 per group) and day 7 (P7) (n = 3 per group). **C**, **D** Expression levels of inflammatory factors *IL-1β, IL-6*, and *TNF-α* in the hippocampus (n = 3 per group). **E** Immunofluorescence staining of hippocampal CA3 regions in P7 and P28 mice. Neurons were labeled with NeuN (red), and microglia were labeled with Iba1 (green). The nuclei were stained with DAPI (blue). Scale bars = 50 µm. **F** The quantification of NeuN positive cells in the hippocampus at P7 (n = 3 per group). **G** The quantification of microglial process length at P28 (n = 15 per group). **H** The quantification of microglial branch points at P28 (n = 15 per group). **I** The quantification of microglial endpoints at P28 (n = 15 per group). Data are presented as mean ± SD and *P < 0.05, **P < 0.01 vs. WT using two-way ANOVA followed by Sidak’s multiple comparisons test.

To investigate whether the *GABRG2(A105T)* mutation induces neuroinflammatory changes in the hippocampus independently of seizure activity, we quantified the expression levels of key inflammatory factors in hippocampal tissues of neonatal mice at postnatal day 1 (P1) and day 7 (P7) using qRT-PCR. Notably, at both P1 and P7, the expression of *ccl2* and *ccl5* was significantly elevated in *Gabrg2*^*+/A105T*^ mice compared to WT controls (*P* < 0.01, Fig. 7A, B). In contrast, the expression of *IL-1β* and *IL-6* was increased only at P7, while TNF-α levels were reduced at P1 (*P* < 0.05 or 0.01, Fig. 7C, D). These findings suggested that the *GABRG2(A105T)* mutation induced an early and selective upregulation of specific inflammatory mediators, such as *ccl2* and *ccl5*, in the hippocampus prior to the onset of seizures. The delayed increase in *IL-1β* and *IL-6* at P7 might indicate a progressive inflammatory response, while the reduction in *TNF-α* at P1 could reflected a compensatory mechanism or temporal-specific regulation. These results highlighted that the *GABRG2(A105T)* mutation itself might drive neuroinflammatory changes in the developing hippocampus, potentially contributing to the pathogenesis of epilepsy and neurodevelopmental abnormalities. Further studies are needed to elucidate the precise mechanisms by which these inflammatory factors influence neuronal excitability and epileptogenesis.

To further investigate the impact of the *GABRG2(A105T)* mutation on microglial activation, we performed immunofluorescence staining on hippocampal tissues in P7 and P28 mice. Using red fluorescence to label NeuN-positive neurons and green fluorescence to label Iba1-positive microglia (Fig. 7E), we observed a significant reduction in NeuN-positive neurons in KI mice at P7 (Fig. 7F), which was consistent with the findings in Fig. 4G, revealed, indicating neuronal loss.

To further assess microglial activation, we conducted morphometric analysis of Iba1-immunolabeled cells in the hippocampal region (Fig. 7E). The microglial process length, number of branches, and endpoints were quantified. Compared to WT controls, microglia in *Gabrg2*^*+/A105T*^ mice exhibited significantly reduced process length, fewer branches, and decreased numbers of endpoints, indicating a shift toward an activated, amoeboid morphology (Fig. 7G–I). This morphological transformation of microglia suggests that the *GABRG2(A105T)* mutation induces robust microglial activation and recruitment, potentially contributing to a proinflammatory hippocampal environment. Given the critical role of the hippocampal region in synaptic plasticity and seizure propagation, the presence of activated microglia supports the hypothesis that neuroinflammation associated with the *GABRG2(A105T)* mutation contributes to both neuronal loss and epileptogenesis. These findings underscore the potential involvement of microglial activation in the pathological mechanisms underlying *GABRG2*-related epilepsy.

## Discussion

### Development of a novel mouse model of *GABRG2*-associated DEE

Our *Gabrg2*^*+/A105T*^ knock-in mouse represents the first DEE mouse model based on a missense *GABRG2* mutation, offering new insights into pathogenic mechanisms distinct from those of truncation-based models. This model displays spontaneous generalized seizures, cognitive impairments, and early-onset neuroinflammation—mirroring clinical features observed in patients with *GABRG2*-related DEE.

Several *GABRG2* knock-out (KO) and knock-in mouse models have been generated to investigate genotype-phenotype correlations in *GABRG2*-associated epilepsies. For instance, *Gabrg2*^*+/−*^ KO mice exhibit anxiety with or without absence seizures, modeling mild epilepsy through γ2 subunit haploinsufficiency [21]. *Gabrg2*^*+/R82Q*^ mice display absence seizures/febrile seizure susceptibility, and reduced surface expression and cortical inhibition, but with background-strain-dependent effects. Mechanistically, the two seizure phenotypes are mediated by distinct mechanisms: haploinsufficiency for absence seizures and a “dominant” effect for febrile seizures [22]. *Gabrg2*^*+/K328M*^ mice associated with GEFS+ in humans, present with spontaneous seizures caused by altered receptor gating kinetics despite normal trafficking [23]. *Gabrg2*^*T316N/+*^ mice shared several key phenotypes with patients with sleep-related hypermotor epilepsy, such as spontaneous seizure attacks during sleep and sleep fragmentation, with decreased levels and disrupted circadian variation in membrane and synaptosome expressions of γ2 subunits of the GABA_A_R [24]. In contrast, *Gabrg2*^*+/Q390X*^ mice model severe epilepsy, with spontaneous tonic-clonic seizures, myoclonic jerks, SUDEP, and profound behavioral abnormalities. Mechanistically, the Q390X truncation leads to ER retention and cytoplasmic accumulation of misfolded γ2 subunits, triggering ER stress and elevated total γ2 protein levels.

Uniquely, A105T results in reduced total γ2 expression, especially in the hippocampus, likely due to impaired subunit folding or trafficking. Electrophysiological recordings reveal diminished mIPSC amplitude in CA1 pyramidal neurons, indicating weakened synaptic inhibition. The structural modeling is consistent with the possibility that reduced γ2 expression may reflect decreased protein stability; however, additional biochemical studies (e.g., pulse-chase assays) are required to test this hypothesis.

Together, these models define a mechanistic continuum: from haploinsufficiency (R82Q and T316N), gating dysfunction (K328M), to dominant-negative retention (Q390X), and now-via A105T-region-specific reduction in receptor expression caused by a missense mutation. The contrasting patterns of γ2 protein expression—increased in Q390X vs decreased in A105T—highlight diverse but converging mechanisms driving GABAergic dysfunction and epileptogenesis in *GABRG2*-related DEE.

### Contribution of the *GABRG2(A105T)* mutation to epileptic seizures in mice

Multiple mutations in *GABRG2* have been associated with epilepsy syndromes with different severities. Some mutations in *GABRG2* are associated with simple febrile seizures or CAE, with good outcomes, while others are associated with the more severe GEFS+ phenotype, which continues into adulthood. A subset of children has a more severe disease phenotype, including DEE. One such mutation is A106T, located in the N-terminal (β1-β2 loop). To date, nine cases of epilepsy patients harboring the *GABRG2 (A106T)* missense mutation have been reported (Table 1). We show that *Gabrg2*^*+/A105T*^ mice displayed spontaneous generalized tonic-clonic seizures as early as P14.

**Table 1:** Overview of reported cases with *GABRG2(A106T)* mutations.

Patient No	1 [19]	2 [19]	3 [28]	4 [28]	5 [28]	6 [28]	7 [29]	8 [29]	9 [50]
Sex	Female	Male	Female	Male	Female	Female	Female	Female	Female
Age at seizure onset	Day 1	3 months	4 months	6 weeks	Day 1	Day 2	Day 40	Day 2	
Seizure types	GTCS,tonic, FS	Tonic, FS, atonic	GTCS, atonic	GTCS, FS	GTCS, FS, myoclonic	GTCS, FS, myoclonic	FS	FS; GTCS, SE	
AED responses	LEV	No clear response	No clear response	No clear response	Pyridoxine OXC	LEV; OXC	VPA; OXC	TPM; LEV	No clear response
Seizure Outcomes	Seizure-free	Intractable	Intractable	Intractable	Intractable	Seizure-free	Seizure-free	Seizure-free	Intractable
MRI findings	Delayed myelination	Volume loss	Normal	Normal	Normal	Normal	Cortex dysplasia, delayed myelination	Normal	
Visual impairment	No	No	Yes	No	Yes	Yes	No	No	
Development	Delay	Delay	Delay	Delay	Delay	Delay	Delay	Delay	

Underlining indicates treatment with clinical response.

*GTCS* generalized tonic-clonic seizure, *FS* focal seizure, *SE* status epilepticus, *LEV* levetiracetam, *VPA* valproic acid, *TPM* topiramate, *OXC* oxcarbazepine.

Due to a mild ~30% reduction in current density of heteromeric GABA_A_ receptor channels, haploinsufficiency is proposed to mediate DEE caused by the *GABRG2(A105T)* variant. The results from in vitro studies suggested an underlying common molecular and functional basis, reduction of GABAergic synaptic inhibition. All *GABRG2* mutations, to different extents, reduced GABA_A_ receptor channel function by diverse mechanisms including impaired expression, assembly, trafficking, GABA binding, and channel gating [25]. The basis for the severity of epilepsy phenotypes with GABR mutations is likely related to the extent of reduction of receptor function. However, heterozygous *Gabrg2* KO mice display only anxiety [21] and are reported to have absence seizures in some genetic backgrounds [22]. These differences suggest that the seizure phenotypes in *Gabrg2*^*+/A105T*^ mice do not occur as a simple consequence of the loss of one *GABRG2* allele.

### Effects of the *GABRG2(A105T)* mutation on cognition and behavior of mice

Patients with the A106T mutation had profound intellectual disability and autistic behavior [19]. Similarly, the introduction of the p.(A105T) variant resulted in cognitive disorders with regard to spatial learning and memory in *Gabrg2*^*+/A105T*^ mice. They performed poorly on the Morris water maze test, a task designed to assess hippocampus-dependent spatial memory. These results support the implication of p. (A105T) in the hippocampus functions in lines with previous conclusions. Similar hippocampus-dependent spatial memory deficits were reported in the heterozygous *Gabrg2*^*+/Q390X*^ mice [26]. The *Gabrg2*^*+/A105T*^ mice did not show deficits in nonspatial memory of object identity in new object recognition test, which relies on the perirhinal cortex and to a lesser extent on the hippocampus [4]. Neuronal degeneration and neuroinflammation in the hippocampus may underlie these memory deficits in *Gabrg2*^*+/A105T*^ mice.

Interestingly, the heterozygous *Gabrg2*^*+/A105T*^ mice do not exhibit locomotor hyperactivity. Heterogenous *Gabrg2*^*+/-*^ constitutive knockout mice [21] and neocortex- and hippocampus-specific deletion of *Gabrg2* exhibited anxiety [27]. Comparison between *Gabrg2*^*+/A105T*^ mice and *Gabrg2*^*+/Q390X*^ mice revealed that both genotypes show anxiety. We speculate that anxiety may likely be due to the heterozygous loss of *Gabrg2* in the hippocampus.

### Neuropathology in mouse hippocampus induced by *GABRG2(A105T)*

The present study provides evidence that the expression of mutant *GABRG2(A105T)* induces neurodegeneration in the hippocampus of mice. These findings are consistent with dysplasia of the frontal and temporal cortex in two patients carrying the A106T mutation [28, 29]. Both of them had intractable seizures. The hippocampus plays an important role in generating behavioral seizures. In mesial temporal lobe epilepsy, hippocampal sclerosis is closely associated with drug-resistant seizures, progressive cognitive decline, high risk of mortality, and sudden unexpected death in epilepsy [30].

It is noteworthy that neuroinflammation was observed in *Gabrg2*^*+/A105T*^ mice and *Gabrg2*^*+/Q390X*^ mice but not *Gabrg2*^*+/−*^ KO mice, even though only *GABRG2(Q390X)* mutation caused dominant negative suppression of GABA_A_ receptors [31]. These differences may be related to different “mutant over WT” expression ratios, which dictate the extent of dominant-negative suppression of the pentameric GABA_A_ channel. However, these differences also highlight the complexity and heterogeneity of the pathophysiology associated with each mutation. Other unique effects besides dominant negative effects may contribute to neurodegeneration.

Although epileptic seizures are thought to cause cognitive and behavioral impairments in DEE [32], our findings suggest that severe seizure phenotypes, reduced viability, and cognitive deficits seen in *Gabrg2*^*+/A105T*^ mice may be due to neurodegeneration and neuroinflammation in addition to hyperexcitability in the hippocampus.

### Increased neuroinflammation in mouse hippocampus induced by *GABRG2(A105T)*

Neuroinflammation is a physiological response to preserve homeostasis; however, when chronically sustained or excessively exacerbated, it can lead to neurodegeneration and the development of epilepsy [33]. Accumulating evidence suggests that neuroinflammation is not merely a consequence of seizures or brain damage but may actively participate in their pathogenesis [33, 34]. Upregulation of proinflammatory genes involved in age-dependent neuroinflammation has been documented in the *Gabrg2*^*+/Q390X*^ mice mouse model of Dravet syndrome before seizure onset [35]. Similarly, *Gabrg2*^*+/A105T*^ mice display neuroinflammatory activation prior to the development of spontaneous seizures, supporting a convergent inflammatory mechanism.

In the current study, we observed reactive microgliosis in the hippocampus of both newborn and adult *Gabrg2*^*+/A105T*^ mice compared to aged-matched WT controls. While our current analysis focused on the hippocampus, we acknowledge that neuroinflammation may not be confined to this region. Thus, the hippocampal changes reported here should be viewed as regionally focused findings and may serve as a starting point for future investigations aimed at determining whether neuroinflammation in *Gabrg2*^*+/A105T*^ mice is restricted to specific areas or reflects a more global process. The *Gabrg2*^*+/A105T*^ mouse model may therefore provide a valuable platform for studying neuroinflammatory mechanisms in DEE and for evaluating the efficacy of anti-inflammatory drugs.

Importantly, neuroinflammation in *Gabrg2*^*+/A105T*^ mice appears to precede seizure onset. Proinflammatory cytokines, including IL-1β and IL-6, were significantly elevated in the hippocampus by P14, whereas spontaneous seizures are first detected more than two weeks later. Additionally, increased expression of chemokines Ccl2 and Ccl5 was evident at P1 and P7. The C-C motif chemokine ligand (CCL) family is one of the core contributors to neuroinflammation and plays a pivotal role in seizure progression. CCL2 and CCL5 are upregulated in the hippocampus and other temporal lobe regions of patients with seizures [36, 37]. Chemokines interact with chemokine receptors to exert their biological function. CCL2/CCR2 signaling is required for resident microglial activation and monocyte infiltration, which in turn contributes to kainic acid (KA)-induced neuronal loss [38]. CCR5 has emerged as a significant player in the inflammatory processes associated with a spectrum of brain diseases, including epilepsy, stroke, Alzheimer’s disease, and multiple sclerosis [39]. In KA-treated mice, CCL5 is highly expressed in activated microglia and astrocytes in the hippocampus, promotes cell migration via CCR5. Notably, targeting the CCL5/CCR5 axis with the FDA-approved CCR5 antagonist maraviroc has been shown to attenuate neuroinflammation following seizures and confer therapeutic effects [40, 41].

Given that the *GABRG2(A105T)* mutation is present from conception, we speculate that neuroinflammation may emerge during embryonic development. Chronic accumulation of mutant γ2 subunits within host cells may overwhelm protein degradation pathways, depending on the cell capacity of protein disposal. This may partially explain why spontaneous seizures develop in *Gabrg2*^*+/A105T*^ mice but not in *Gabrg2*^*+/−*^ mice, in which no mutant protein is produced.

It is unclear how expression of *GABRG2(A105T)* mutation results in neurodegeneration in vivo. It has been hypothesized that in *Gabrg2*^*+/Q390X*^ mice, intracellular retention of *GABRG2(Q390X)* proteins in the ER and their continuous clearance could saturate the capacity of the ER and proteasome, leading to apoptosis. However, in *Gabrg2*^*+/A105T*^ mice, we did not observe overt intracellular aggregation of mutant γ2 proteins. Further studies are warranted to elucidate how mutant γ2 expression disrupts neuronal integrity and to determine the relative contributions of neuroinflammation, GABA_A_ receptor dysfunction, and/or chronic ER stress to the development of refractory seizures in this DEE model.

## Methods

### Generation of transgenic knock-in mice

G*abrg2*^*+/A105T*^ mice were generated on the C57BL/6J background via gene targeting by Cyagen Biosciences (Soochow, China). The mouse *Gabrg2* gene (GenBank accession number: NM_008073.4; Ensembl: ENSMUSG00000020436) is located on mouse chromosome 11. The p. A105T (GCT to ACT) point mutation was introduced into exon 3 in 3′ homology arm. The targeting vector was electroporated into mouse embryonic stem cells (ESCs), and the positive clone was injected into C57BL/6 albino embryos, which were then re-implanted into pseudo-pregnant females. Founder animals were identified by their coat color. Their germline transmission was confirmed by breeding with C57BL/6 females and subsequent genotyping of the offspring. Genotyping was performed using genomic DNA prepared from ear punch biopsies with Quick Genotyping Assay Kit for Mouse Tail (Beyotime, Shanghai, China) and the genotyping primer were the following: Forward-GGTCATTGGGGAAGATTTTGATTCG, Reverse-CGGAAGCTGTTTGTGATTCCTGAC. All animal experiments were approved by the Administration Committee of Experimental Animals of Nantong University, China (No. P20230303-001). Animals were assigned to experimental groups based on genotype, and no randomization procedure was used.

### Structural modeling

We utilized the high-resolution cryo-EM structure of the human pentameric GABA_A_ receptor (PDB: 6HUO [16]) in combination with DynaMut [42] to predict the changes in stability before and after mutations in the γ2 subunit. The results from the point mutation simulations included ΔΔGstability for the mutant. Negative values indicate decreased stability, and positive values indicate increased stability. We used Chai-1 in combination with MMSeq2 [43] to predict the structural models of the mutants, selecting the model with the highest confidence from the five candidates for structural analysis. Structural alignment was performed in UCSF Chimera [44], and interactions were considered possible if the atomic distance between residues was less than 4 Å.

### Video recording and seizure score

The *Gabrg2*^*+/A105T*^ (A105T) mice and the wild-type (WT) littermates at 46 months were placed in transparent glass container and acclimate to the environment for 2 h prior a 2-h video recording. Seizure severity was identified according to the modified Racine scale as previously described. Briefly, Stage 1: immobility or flattening; Stage 2: rigid posture with forelimb or tail extension; Stage 3: continuous head bobbing; Stage 4: rearing and falling; Stage 5: continuous rearing and falling; Stage 6: loss of posture and generalized convulsive activity. To assess the seizure threshold, pentylenetetrazol (PTZ) (40 mg/kg) was administrated by intraperitoneal injection and subjected to a 30-min video recording subsequently. The seizure severity was evaluated by the modified Racine scale, and the latency to seizure onset and duration of seizures were recorded. The final score was the mean rating from two independent researchers.

### Whole-cell patch-clamp recordings of miniature inhibitory postsynaptic currents (mIPSC) in hippocampal CA1 pyramidal neurons

Mice (postnatal day P28, as specified) were deeply anesthetized with isoflurane (3–5% in oxygen) and decapitated. The brain was rapidly removed and immersed in ice-cold N-methyl-D-glucamine (NMDG)-based cutting solution containing (in mM): 92 NMDG, 2.5 KCl, 1.25 NaH₂PO₄·H₂O, 20 HEPES, 2 Thiourea.5 Na-ascorbate, 3 Na-pyruvate, 10 MgSO₄·7H₂O, 30 NaHCO₃, 25 glucose, and 0.5 CaCl₂, bubbled continuously with 95% O₂/5% CO₂ (pH 7.3–7.4, osmolarity 300–310 mOsm). Coronal brain slices (300 μm thick) containing the hippocampal region were cut using a vibrating blade microtome (Leica VT1200S; Wetzlar, Germany). Slices were transferred to a holding chamber filled with artificial cerebrospinal fluid (aCSF) containing (in mM): 124 NaCl, 2.5 KCl, 1.25 NaH₂PO₄·H₂O, 2 MgSO₄·7H₂O, 24 NaHCO₃, 5 HEPES, 12.5 glucose, and 2 CaCl₂, bubbled with 95% O₂/5% CO₂. Slices were incubated at 32 °C for 30–45 min to recover and then maintained at room temperature (22–25 °C) for at least 1 h before recording.

Each slice was transferred to a submerged recording chamber continuously perfused with aCSF (bubbled with 95% O₂/5% CO₂) at a flow rate of 2–3 mL/min. The chamber was mounted on an upright microscope (Olympus BX51WI, Tokyo, Japan) equipped with infrared differential interference contrast (IR-DIC) optics and a 40× water-immersion objective. Patch pipettes were pulled from borosilicate glass capillaries (1.5 mm outer diameter, 0.86 mm inner diameter; Sutter Instruments, Novato, CA, USA) using a horizontal puller (P-1000, Sutter Instruments) to a final resistance of 3–5 MΩ. The internal pipette solution contained (in mM): 130 CsCl, 10 4-(2-Hydroxyethyl)-1-piperazineethanesulfonic acid (HEPES), 10 EGTA, 2 Mg-ATP, 0.3 Na-GTP, and 5 QX-314 (lidocaine N-ethyl bromide), adjusted to pH 7.2–7.3 with CsOH and osmolarity of 285–295 mOsm. The inhibitory current appeared as inward currents at a holding potential of −70 mV. Miniature inhibitory postsynaptic currents (mIPSC) were recorded in voltage-clamp mode at a holding potential of −70 mV using a Multiclamp 700B amplifier (Molecular Devices, San Jose, CA). To isolate mIPSC, the perfusate was supplemented with tetrodotoxin (TTX, 1 μM) to eliminate action potential-dependent synaptic activity, 2,3-dioxo-6-nitro-1,2,3,4-tetrahydrobenzo[f]quinoxaline-7-sulfonamide (NBQX) (50 μM) to block α-Amino-3-hydroxy-5-methyl-4-isoxazolepropionic acid (AMPA) receptors, and AP-5 (50 μM) to block N-Methyl-D-aspartic acid (NMDA) receptors. Signals were low-pass filtered at 2 kHz and digitized at 10 kHz using a Digidata 1550 A interface (Molecular Devices) controlled by pClamp 11.2 software (Molecular Devices). Recordings were included for analysis only if the series resistance (Rs) remained stable (<20% change) throughout the recording period and did not exceed 20 MΩ, the holding current was <200 pA to minimize space-clamp errors, mIPSC events were clearly distinguishable from baseline noise (amplitude >5 pA), and each neuron was recorded for 5–10 min in gap-free mode after achieving a stable whole-cell configuration (≥5 min post-patch rupture).

### Electroencephalography (EEG) recording

Subdural electrodes using stainless steel screws and multi-stranded copper wire were surgically implanted in the *Gabrg2*^*+/A105T*^ mice. To prevent retinal inflammation in mice, erythromycin was administered prior to the implantation of the electrode. A 1.5 cm incision was made along the midline of the skull following disinfection procedures. Three holes were created in the dura mater with a small handheld craniotomy drill. The first hole was positioned 1–2 mm left and anterior to the Bregma area, while the other two holes were located on the posterior side of Bregma, outside of the central sulcus by 2–3 mm. The subdural electrodes were implanted into the designated holes and dental acrylic cement were applied to reinforce the stability of electrodes. Seven days post-implantation, EEG was recorded for a duration of 30 min using the MD3000 Bio-signal Acquisition and Processing System [45] (version 2.0, Anhui Zhenghua). Additionally, EEG recordings were obtained following the intraperitoneal administration of PTZ for another 30-min period.

### Behavioral analysis

All experiments were conducted using mice aged 4–6 months (equal numbers of males and females) unless otherwise specified. All mice underwent acclimation prior to testing. Investigators were blinded to group allocation throughout the experiment and during all outcome assessments.

### Morris water maze (MWM)

The MWM test was performed to measure the learning and spatial memory of the knock-in mice. The circular tank is 150 cm in diameter and 60 cm in depth, filled with water at 22 °C. It’s divided into four equal quadrants and the platform is 10-cm diameter positioned at the center of the first quadrant, which was covered up 2 cm beneath the water’s surface. Fixed visual cues were pasted onto the walls around the pool to provide visual cues to the mice during the experiment. During the learning phase, the mice were trained four times a day at fixed intervals, with a 15-min gap between each training session. This training continued for four consecutive days. On the first day, place the mice from the first to fourth quadrants along the pool wall, ensuring they are facing away from it before immersing them in water. Observe and record their behavior for 60 s. If a mouse located the platform within this time frame, timing will cease; however, the experiment concludes only when the mouse remains on the platform for 10 s. Should a mouse find the platform but leave before staying on it for 10 s, timing will continue until a total observation period of 60 s is reached. In cases where a mouse failed to locate the platform independently, assistance will be provided by guiding it towards its location. On the second day of training, position the mice in sequence within the second, first, fourth, and third quadrants along the pool wall while maintaining their orientation away from it prior to immersion in water. The third day’s procedure involves placing them in order within quadrants three through one (third quadrant followed by fourth quadrant then first and finally second), again facing away from any walls during immersion. Finally, on day four of training sessions arrange them sequentially starting with quadrant four down to one (fourth quadrant followed by third then second and concluding with first) while ensuring they remain oriented away from walls as they are immersed. During the testing procedures, remove all platforms entirely and position each mouse solely within quadrant three along that same pool wall while keeping them directed away from said wall. The activity of each mouse was observed over an uninterrupted duration of 60 s. The total distance traveled, mean swimming speed, number of platform entries and latency to the first entry of platform were recorded. The behavioral data were processed with ANY-maze (version 7.2, Stoelting).

### Open field test

The open-field arena is a 40 × 40 × 40 cm (width × length × height) cubical enclosure. The mouse was placed in a corner of the box and recorded by an overhead video camera for 20 min. The total distance traveled, average motor speed, and frequency of entries into the central area for each mouse were analyzed [46].

### Elevated plus maze

Before the experiment, the mice were acclimated to the testing environment for 24 h. The elevated plus maze device contains two open arms and two closed arms (100 cm long, 3.6 cm large), elevated 80 cm from the floor. The mice were taken out of the cage and gently placed face down in the central area of the apparatus, with the head towards the enclosed arm. The movements of each mouse were tracked and recorded for a 5-min period. The duration spent in the open and closed arms, the number of entries into the open and closed arms, as well as the average speed within these respective areas were analyzed.

### New object recognition

The experimental chamber was maintained in a clean and odor-free condition. Before initiating the training phase, objects A and B were positioned at the left and right ends of one side wall, respectively, while the mice were placed with their backs facing these two objects within the arena. The interactions between the mice and both objects were recorded over a period of 5 min. Following this training session, the mice were allowed to rest for 1 h before proceeding to the test phase. During testing, object B was substituted with object C in the arena; however, the mice remained positioned with their backs towards both objects. The exploratory behavior exhibited by the mice towards object C was documented for an additional 5 min. The total number of explorations, distance traveled, and duration spent exploring both new and old objects were subsequently calculated.

### Nissl staining

After the behavioral tests, the mice were transcardial perfusion with saline, followed by fixation in 4% paraformaldehyde in 0.1 M PBS. The brains were subsequently dissected and underwent a series of processing steps that included washing in water, dehydration through a graded ethanol series, clearing in xylene, embedding in paraffin, and sectioning into slices of 5 µm thickness. Nissl staining was conducted on these sections prior to capturing images using a Leica microscope (Leica Microsystems, Wetzlar, Germany) as previously reported. A total of three mice from each group were utilized for the experiments.

### Western blot analysis

The total protein and the membrane protein of the whole brain tissues, cortex, hippocampus, thalamus and cerebellum were separated from the WT and A105T mice respectively. Protein quantification was performed using BCA analysis, followed by separation via SDS-PAGE electrophoresis. After transferring the proteins to a PVDF membrane (Millipore, Bedford, MA), the membrane was blocked with 5% nonfat dry milk in Tris-buffered saline (TBS; pH 7.4) and incubated overnight at 4 °C with rabbit anti-GABRG2 antibody (Synaptic System, #224003; 1:500), rabbit anti-GAPDH (Sangon Biotech, #D110016; 1:1000), and mouse anti-ATPase (Abcam, #ab76020, 1:1000). GAPDH and ATPase served as internal controls for total protein and membrane protein analyses, respectively. Following washes with TBS/T (TBS containing 0.1% Tween 20), HRP-conjugated affinity purified goat anti-rabbit IgG H&L (Abcam, #ab6721, 1:5000) or goat anti-mouse IgG H&L (Abcam, #ab6789, 1:5000) was applied at room temperature for 30 min. The membrane was incubated with enhanced chemiluminescence (ECL) substrate and subsequently scanned using an imaging system (Tanon, Shanghai, China). The resulting images were analyzed utilizing ImageJ software version 1.48 (Bethesda, MD, USA).

### RNA sequencing and qRT-PCR

The RNA sequencing and data analysis were conducted by GENEWIZ (Suzhou, China), as previously described. In brief, hippocampal samples from WT and A105T mice underwent RNA extraction, yielding a total of 1 μg of RNA with a RIN value exceeding 6.5 for subsequent library preparation and transcriptome sequencing. This was performed using the HiSeq Control Software (HCS) + OLB + GAPipeline-1.6 (Illumina) on the HiSeq instrument. Gene sets with a p < 0.05 and a false discovery rate <0.05 were considered significantly enriched. Gene ontology (GO) analysis and Kyoto Encyclopedia of Genes and Genomes (KEGG) pathway analysis were carried out as previously reported. The expression of some differentially expressed genes (DEGs) in the hippocampus was validated by qRT-PCR as previously described [47] with a StepOne real-time PCR system (Applied Biosystems, Foster City, CA) and the primer used are listed in Table 2. The relative mRNA expression levels were calculated using the comparative ΔΔCt method and normalized against GAPDH.

**Table 2:** Oligonucleotide primers used in qRT-PCR.

Gene	Primer sequence (5′- 3′)
*gapdh*	Forward-AACTTTGGCATTGTGGAAGG
	Reverse-ACACATTGGGGGTAGGAACA
*gm49339*	Forward-CCCACTGAAGATGGACTGG
	Reverse-TTGTGGGTTCCAACTGTTCA
*clec4d*	Forward-GCTGGAAGAATCCCAAATGA
	Reverse-ACTTCCTCTCGTCCAGCGTA
*ifi205*	Forward-TCCACAACCCAGGAAGAGAC
	Reverse-GAAGCCGAAGATGAGACCTG
*ccl2*	Forward-AGGTCCCTGTCATGCTTCTG
	Reverse-TCTGGACCCATTCCTTCTTG
*ccl5*	Forward-CCCTCACCATCATCCTCACT
	Reverse-CCTTCGAGTGACAAACACGA
*lilrb4b*	Forward-ACCCACTGAAGATGGACTGG
	Reverse-TGTTCAGCTCTGCATTGTCC
*il-1b*	Forward-GGGCCTCAAGGAAAAGAATC
	Reverse-TTCTGCTTGAGAGGTGCTGA
*oas3*	Forward-GTCAAACCCAAGCCACAAGT
	Reverse-TGTAGGCACACCTGGTGGTA
*cybb*	Forward-TCACTTCCTCCACCAAAACC
	Reverse-GGGATTGGGCATTCCTTTAT
*ifi211*	Forward-ATTCTGGATTGGGCAAACTG
	Reverse-CTCTTCCTGGGTTGCAGAAG

### Immunohistochemistry

The brain tissues of WT and A105T mice were directly dissected and fixed in 4% paraformaldehyde for 30 min before being stored in a 30% sucrose solution. The brains were sectioned using a cryostat at thicknesses of 15 μm and preserved at −20 °C. The sections were permeabilized with 0.4% Triton X-100 for 10 min, followed by blocking with a solution containing 0.2% BSA and 0.2% Triton X-100 for one hour [48]. The sections were incubated with mouse anti-GABRG2 antibody (Synaptic System, #224011; 1:200) and rabbit anti-beta III Tubulin, Mouse (Abcam, #ab7751; 1:200) or NeuN (Abcam, #ab104224, 1:500) and Iba1 (Abcam, #ab178846, 1:500) at 4 °C overnight and after washing with a solution of 0.1% BSA/PBA three times. Then the sections were incubated with the secondary antibody, fluorescein isothiocyanate (FITC)-conjugated rabbit anti-mouse IgG and Cy3-conjugated donkey anti-rabbit IgG (Abcam, #ab6724, #ab97075;1:400) at room temperature for 2 h. Cell nuclei were labeled using Hoechst 33342 (5 μg/mL, Sigma Aldrich, St. Louis, MO, USA) or DAPI (Beyotime Biotechnology), after which they were washed again and mounted directly using CitiFluor (Beyotime Biotechnology). Confocal images were acquired using Zeiss ZEN (Blue edition, version 3.9, Zeiss), and image processing was conducted with Adobe Photoshop (CC 2019).

For microglia skeleton analysis, confocal images (30 μm thick) of Z-stacks from the mouse hippocampal regions were acquired and converted to maximum intensity projections using Zeiss ZEN software and the quantification was performed according to the protocol described previously [49]. Images were converted to binary images using ImageJ Fiji software and intact microglia were selected, skeletonized using the Skeletonize plugin, and analyzed using the AnalyzeSkeleton (2D/3D) plugin (http://imagej.net/AnalyzeSkeleton). The microglial process length, number of branch points and endpoints were quantified and compared.

### Statistical analysis

No statistical methods were used to pre-determine sample sizes; however, group sizes were based on previous studies in the field and are consistent with commonly accepted standards in neurophysiological and behavioral research. No animals or data points were excluded from analysis, except in electrophysiological recordings, where pre-established quality control criteria were applied. Data analysis and processing were carried out with GraphPad Prism (8.0.1, GraphPad Software). All data are presented as mean ± standard error (SD). Unpaired Student’s t-test was employed to compare two groups and two-way analysis of variance (ANOVA), followed by Sidak’s multiple comparisons test was conducted for comparisons involving multiple groups across two factors. Normality was assessed using the Shapiro–Wilk test, and homogeneity of variances was tested using the F-test. For non-normally distributed data, non-parametric tests (Mann–Whitney U test or Kruskal-Wallis test) was applied. A *P* value of less than 0.05 was considered statistically significant.

## Supplementary information

Supplementary figure legends

Supplementary video 1A

Supplementary video 1B

Supplementary Figure 1

Supplementary Figure 2

Supplementary Figure 3

Supplementary Figure 4

original western blots

## Data Availability

Data will be made available on request.

## References

[1] Scheffer IE, Berkovic S, Capovilla G, Connolly MB, French J, Guilhoto L, et al. ILAE classification of the epilepsies: position paper of the ILAE commission for classification and terminology. Epilepsia. 2017;58:512–21. 28276062 10.1111/epi.13709 PMC5386840

[2] Scheffer IE, Zuberi S, Mefford HC, Guerrini R, McTague A. Developmental and epileptic encephalopathies. Nat Rev Dis Prim. 2024;10:61. 39237642 10.1038/s41572-024-00546-6

[3] McTague A, Howell KB, Cross JH, Kurian MA, Scheffer IE. The genetic landscape of the epileptic encephalopathies of infancy and childhood. Lancet Neurol. 2016;15:304–16. 26597089 10.1016/S1474-4422(15)00250-1

[4] Specchio N, Trivisano M, Aronica E, Balestrini S, Arzimanoglou A, Colasante G, et al. The expanding field of genetic developmental and epileptic encephalopathies: current understanding and future perspectives. Lancet Child Adolesc Health. 2024;8:821–34. 39419567 10.1016/S2352-4642(24)00196-2

[5] Noebels J. Pathway-driven discovery of epilepsy genes. Nat Neurosci. 2015;18:344–50. 25710836 10.1038/nn.3933 PMC4852130

[6] Epi PMC. A roadmap for precision medicine in the epilepsies. Lancet Neurol. 2015;14:1219–28. 26416172 10.1016/S1474-4422(15)00199-4 PMC4663979

[7] Epi KC, Epilepsy Phenome/Genome P, Allen AS, Berkovic SF, Cossette P, Delanty N, et al. De novo mutations in epileptic encephalopathies. Nature. 2013;501:217–21. 23934111 10.1038/nature12439 PMC3773011

[8] Chua HC, Chebib M. GABA(A) Receptors and the diversity in their structure and pharmacology. Adv Pharm. 2017;79:1–34. 10.1016/bs.apha.2017.03.003 28528665

[9] Maljevic S, Moller RS, Reid CA, Perez-Palma E, Lal D, May P, et al. Spectrum of GABAA receptor variants in epilepsy. Curr Opin Neurol. 2019;32:183–90. 30664068 10.1097/WCO.0000000000000657

[10] Absalom NL, Lin SXN, Liao VWY, Chua HC, Moller RS, Chebib M, et al. GABA(A) receptors in epilepsy: elucidating phenotypic divergence through functional analysis of genetic variants. J Neurochem. 2024;168:3831–52. 37621067 10.1111/jnc.15932 PMC11591409

[11] Hortnagl H, Tasan RO, Wieselthaler A, Kirchmair E, Sieghart W, Sperk G. Patterns of mRNA and protein expression for 12 GABAA receptor subunits in the mouse brain. Neuroscience. 2013;236:345–72. 23337532 10.1016/j.neuroscience.2013.01.008 PMC3605588

[12] Bryson A, Reid C, Petrou S. Fundamental neurochemistry review: GABA(A) receptor neurotransmission and epilepsy: principles, disease mechanisms and pharmacotherapy. J Neurochem. 2023;165:6–28. 36681890 10.1111/jnc.15769

[13] Hernandez CC, Macdonald RL. A structural look at GABA(A) receptor mutations linked to epilepsy syndromes. Brain Res. 2019;1714:234–47. 30851244 10.1016/j.brainres.2019.03.004

[14] Vogel FD, Krenn M, Westphal DS, Graf E, Wagner M, Leiz S, et al. A de novo missense variant in GABRA4 alters receptor function in an epileptic and neurodevelopmental phenotype. Epilepsia. 2022;63:e35–e41. 35152403 10.1111/epi.17188 PMC9304230

[15] Mohammadi NA, Ahring PK, Yu Liao VW, Chua HC, Ortiz de la Rosa S, Johannesen KM, et al. Distinct neurodevelopmental and epileptic phenotypes associated with gain- and loss-of-function GABRB2 variants. EBioMedicine. 2024;106:105236. 38996765 10.1016/j.ebiom.2024.105236 PMC11296288

[16] Absalom NL, Liao VWY, Johannesen KMH, Gardella E, Jacobs J, Lesca G, et al. Gain-of-function and loss-of-function GABRB3 variants lead to distinct clinical phenotypes in patients with developmental and epileptic encephalopathies. Nat Commun. 2022;13:1822. 35383156 10.1038/s41467-022-29280-x PMC8983652

[17] Ahring PK, Liao VWY, Gardella E, Johannesen KM, Krey I, Selmer KK, et al. Gain-of-function variants in GABRD reveal a novel pathway for neurodevelopmental disorders and epilepsy. Brain. 2022;145:1299–309. 34633442 10.1093/brain/awab391 PMC9630717

[18] Musto E, Liao VWY, Johannesen KM, Fenger CD, Lederer D, Kothur K, et al. GABRA1-related disorders: from genetic to functional pathways. Ann Neurol. 2023;95:27–41. 10.1002/ana.26774 37606373

[19] Shen D, Hernandez CC, Shen W, Hu N, Poduri A, Shiedley B, et al. De novo GABRG2 mutations associated with epileptic encephalopathies. Brain. 2017;140:49–67. 27864268 10.1093/brain/aww272 PMC5226060

[20] Walf AA, Frye CA. The use of the elevated plus maze as an assay of anxiety-related behavior in rodents. Nat Protoc. 2007;2:322–8. 17406592 10.1038/nprot.2007.44 PMC3623971

[21] Crestani F, Lorez M, Baer K, Essrich C, Benke D, Laurent JP, et al. Decreased GABAA-receptor clustering results in enhanced anxiety and a bias for threat cues. Nat Neurosci. 1999;2:833–9. 10461223 10.1038/12207

[22] Reid CA, Kim T, Phillips AM, Low J, Berkovic SF, Luscher B, et al. Multiple molecular mechanisms for a single GABAA mutation in epilepsy. Neurology. 2013;80:1003–8. 23408872 10.1212/WNL.0b013e3182872867 PMC3653202

[23] Qu S, Zhou C, Howe R, Shen W, Huang X, Catron M, et al. The K328M substitution in the human GABA(A) receptor gamma2 subunit causes GEFS+ and premature sudden death in knock-in mice. Neurobiol Dis. 2021;152:105296. 33582225 10.1016/j.nbd.2021.105296 PMC8243844

[24] Jiang YL, Xia L, Zhao JJ, Zhou HM, Mi D, Wang X, et al. Mice harboring the T316N variant in the GABA(A)R gamma(2) subunit exhibit sleep-related hypermotor epilepsy phenotypes and hypersynchronization in the thalamocortical pathway. Exp Neurol. 2024;376:114775. 38604438 10.1016/j.expneurol.2024.114775

[25] Macdonald RL, Kang JQ, Gallagher MJ. Mutations in GABAA receptor subunits associated with genetic epilepsies. J Physiol. 2010;588:1861–9. 20308251 10.1113/jphysiol.2010.186999 PMC2901974

[26] Warner TA, Shen W, Huang X, Liu Z, Macdonald RL, Kang JQ. Differential molecular and behavioural alterations in mouse models of GABRG2 haploinsufficiency versus dominant negative mutations associated with human epilepsy. Hum Mol Genet. 2016;25:3192–207. 27340224 10.1093/hmg/ddw168 PMC5179921

[27] Li X, Guo S, Xu S, Chen Z, Wang L, Ding J, et al. Neocortex- and hippocampus-specific deletion of Gabrg2 causes temperature-dependent seizures in mice. Cell Death Dis. 2021;12:553. 34050134 10.1038/s41419-021-03846-x PMC8163876

[28] Zou F, McWalter K, Schmidt L, Decker A, Picker JD, Lincoln S, et al. Expanding the phenotypic spectrum of GABRG2 variants: a recurrent GABRG2 missense variant associated with a severe phenotype. J Neurogenet. 2017;31:30–36. 28460589 10.1080/01677063.2017.1315417 PMC6169784

[29] Yang Y, Niu X, Cheng M, Zeng Q, Deng J, Tian X, et al. Phenotypic spectrum and prognosis of epilepsy patients with GABRG2 variants. Front Mol Neurosci. 2022;15:809163. 35359574 10.3389/fnmol.2022.809163 PMC8964129

[30] Cendes F, Sakamoto AC, Spreafico R, Bingaman W, Becker AJ. Epilepsies associated with hippocampal sclerosis. Acta Neuropathol. 2014;128:21–37. 24823761 10.1007/s00401-014-1292-0

[31] Shen W, Poliquin S, Macdonald RL, Dong M, Kang JQ. Endoplasmic reticulum stress increases inflammatory cytokines in an epilepsy mouse model Gabrg2(+/Q390X) knockin: a link between genetic and acquired epilepsy? Epilepsia. 2020;61:2301–12. 32944937 10.1111/epi.16670 PMC7918935

[32] Berg AT, Berkovic SF, Brodie MJ, Buchhalter J, Cross JH, van Emde Boas W, et al. Revised terminology and concepts for organization of seizures and epilepsies: report of the ILAE commission on classification and terminology, 2005–2009. Epilepsia. 2010;51:676–85. 20196795 10.1111/j.1528-1167.2010.02522.x

[33] Vezzani A, Balosso S, Ravizza T. Neuroinflammatory pathways as treatment targets and biomarkers in epilepsy. Nat Rev Neurol. 2019;15:459–72. 31263255 10.1038/s41582-019-0217-x

[34] Liang W, Wang J, Sui J, Yun F, Shen Y, Zhou J, et al. Inflammation as a target for the treatment of fever-associated epilepsy in zebrafish larvae. Int Immunopharmacol. 2023;116:109802. 10.1016/j.intimp.2023.109802 36738682

[35] Chen H-X, Liang F-C, Gu P, Xu B-L, Xu H-J, Wang W-T, et al. Exosomes derived from mesenchymal stem cells repair a Parkinson’s disease model by inducing autophagy. Cell Death Dis. 2020;11:288. 32341347 10.1038/s41419-020-2473-5 PMC7184757

[36] Kan AA, de Jager W, de Wit M, Heijnen C, van Zuiden M, Ferrier C, et al. Protein expression profiling of inflammatory mediators in human temporal lobe epilepsy reveals co-activation of multiple chemokines and cytokines. J Neuroinflamm. 2012;9:207. 10.1186/1742-2094-9-207 PMC3489559 22935090

[37] Arisi GM, Foresti ML, Katki K, Shapiro LA. Increased CCL2, CCL3, CCL5, and IL-1beta cytokine concentration in piriform cortex, hippocampus, and neocortex after pilocarpine-induced seizures. J Neuroinflamm. 2015;12:129. 10.1186/s12974-015-0347-z PMC4509848 26133170

[38] Tian DS, Peng J, Murugan M, Feng LJ, Liu JL, Eyo UB, et al. Chemokine CCL2-CCR2 signaling induces neuronal cell death via STAT3 activation and IL-1beta production after status epilepticus. J Neurosci. 2017;37:7878–92. 28716963 10.1523/JNEUROSCI.0315-17.2017 PMC5559763

[39] Lin Y, Liu S, Sun Y, Chen C, Yang S, Pei G, et al. CCR5 and inflammatory storm. Ageing Res Rev. 2024;96:102286. 38561044 10.1016/j.arr.2024.102286

[40] Joy MT, Ben Assayag E, Shabashov-Stone D, Liraz-Zaltsman S, Mazzitelli J, Arenas M, et al. CCR5 is a therapeutic target for recovery after stroke and traumatic brain injury. Cell. 2019;176:1143–57.e1113. 30794775 10.1016/j.cell.2019.01.044 PMC7259116

[41] Zhang Z, Li Y, Jiang S, Shi FD, Shi K, Jin WN. Targeting CCL5 signaling attenuates neuroinflammation after seizure. CNS Neurosci Ther. 2023;29:317–30. 36440924 10.1111/cns.14006 PMC9804050

[42] Rodrigues CH, Pires DE, Ascher DB. DynaMut: predicting the impact of mutations on protein conformation, flexibility and stability. Nucleic Acids Res. 2018;46:W350–W355. 29718330 10.1093/nar/gky300 PMC6031064

[43] Steinegger M, Soding J. MMseqs2 enables sensitive protein sequence searching for the analysis of massive data sets. Nat Biotechnol. 2017;35:1026–8. 29035372 10.1038/nbt.3988

[44] Pettersen EF, Goddard TD, Huang CC, Couch GS, Greenblatt DM, Meng EC, et al. UCSF chimera–a visualization system for exploratory research and analysis. J Comput Chem. 2004;25:1605–12. 15264254 10.1002/jcc.20084

[45] Wang J, Wu W, Wan J, Zhan L, Chen Y, Yun F, et al. Preliminary study on the mechanism of SAHA in the treatment of refractory epilepsy induced by GABRG2(F343L) mutation. Biochem Pharmacol. 2024;227:116449. 10.1016/j.bcp.2024.116449 39053637

[46] Zhang HL, Sun Y, Yau SY, Zhou YM, Song XX, Zhang HT, et al. Synergistic effects of two naturally occurring iridoids in eliciting a rapid antidepressant action by up-regulating hippocampal PACAP signalling. Brit J Pharm. 2022;179:4078–91. 10.1111/bph.15847 35362097

[47] Guo B, Qi M, Luo X, Guo L, Xu M, Zhang Y, et al. GIP attenuates neuronal oxidative stress by regulating glucose uptake in spinal cord injury of rat. CNS Neurosci Ther. 2024;30:e14806. 10.1111/cns.14806 PMC11183929 38887182

[48] Geng Y, Yang J, Cheng X, Han Y, Yan F, Wang C, et al. A bioactive gypenoside (GP-14) alleviates neuroinflammation and blood brain barrier (BBB) disruption by inhibiting the NF-κB signaling pathway in a mouse high-altitude cerebral edema (HACE) model. Int Immunopharmacol. 2022;107:108675. 10.1016/j.intimp.2022.108675 35299003

[49] Young K, Morrison H. Quantifying microglia morphology from photomicrographs of immunohistochemistry prepared tissue using ImageJ. J Vis Exp. 2018;136:57648. 10.3791/57648 PMC6103256 29939190

[50] Yamamoto T, Imaizumi T, Yamamoto-Shimojima K, Lu Y, Yanagishita T, Shimada S, et al. Genomic backgrounds of Japanese patients with undiagnosed neurodevelopmental disorders. Brain Dev. 2019;41:776–82. 31171384 10.1016/j.braindev.2019.05.007

