# Supplementary material for: A knock-in mouse model for GABRG2-related epileptic encephalopathy displays spontaneous generalized seizures and cognitive impairment
Source: Cell Death Discov. 2025 Oct 6;11:443. doi: 10.1038/s41420-025-02759-4 (PMC12501280; doi:10.1038/s41420-025-02759-4)
Supplement: Supplementary file 1 — Supplementary figure legends [file 41420_2025_2759_MOESM1_ESM.docx]

## **Supplementary Figure 1. Histological analysis of** ***Gabrg2^+/A105T^* mice.**

Histological analysis of major organs (heart, liver, spleen, lungs, and kidneys) from *Gabrg2^+/A105T^* and WT mice.

## **Supplementary Figure 2. Additional behavioral data.**

(A) Total distance traveled in the Morris water maze. (B) Swimming velocity in the Morris water maze (n = 8 per group). No significant differences were observed between *Gabrg2^+/A105T^* mice and WT controls. (C) Distance traveled in the open and closed arms of the Elevated Plus Maze (n = 16 per group). (D) Velocity in the open and closed arms of the Elevated Plus Maze (n = 16 per group). Data are presented as mean ± SD and ** *P* < 0.01 vs. WT using two-way ANOVA followed by Sidak's multiple comparisons test.

## **Supplementary Figure 3. Visual Evoked Potential (VEP) of *Gabrg2^+/A105T^* mice.**

Flash Electroretinography (ERG) was recorded from both eyes of WT and *Gabrg2^+/A105T^* mice using a Celeris system. Briefly, mice were dark-adapted overnight and then exposed to flashes of light in a dark room with only a dim red light on. (A) Representative VEP trace of WT and *Gabrg2^+/A105T^* mice. (B) The amplitude of VEP was measured (n = 5 per group).

## **Supplementary Figure 4. PCA analysis of the RNA-seq.**

(A) PCA plot visualizing global transcriptome variation. Points represent individual samples, colored by experimental group (WT: blue; A105T: red). (B) Sample correlation heatmap. Hierarchical clustering depicts pairwise Pearson correlation coefficients (r) between samples.

## **Supplementary Video 1.**

Video recording of spontaneous convulsive seizures in *Gabrg2^+/A105T^* mice. (A) WT mouse. (B) *Gabrg2^+/A105T^* mouse.
