# Supplementary figures and images for: A knock-in mouse model for GABRG2-related epileptic encephalopathy displays spontaneous generalized seizures and cognitive impairment
Source: Cell Death Discov. 2025 Oct 6;11:443. doi: 10.1038/s41420-025-02759-4 (PMC12501280; doi:10.1038/s41420-025-02759-4)

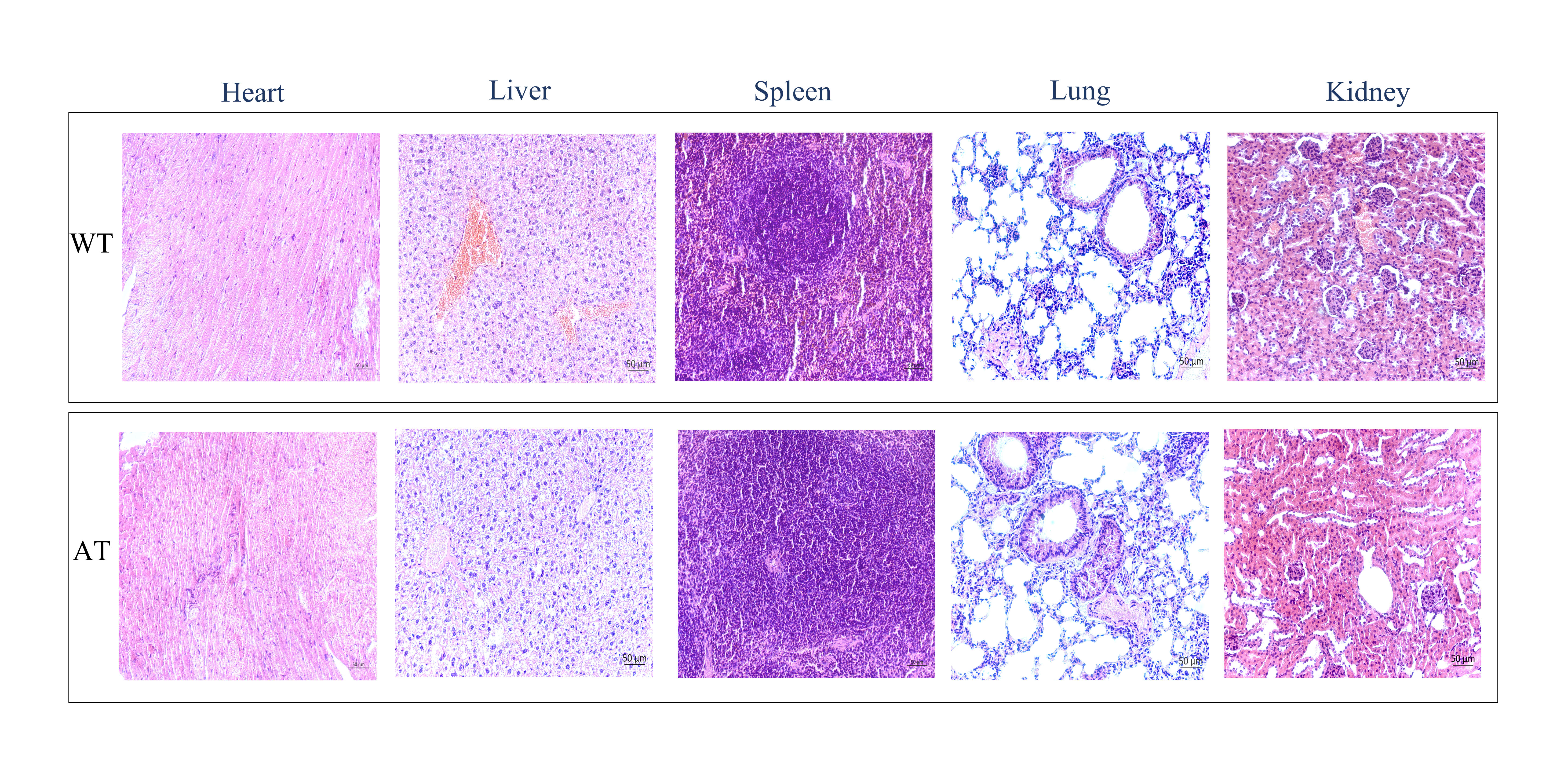

Supplement: Supplementary file 4 — Supplementary Figure 1 [file 41420_2025_2759_MOESM4_ESM.tif]

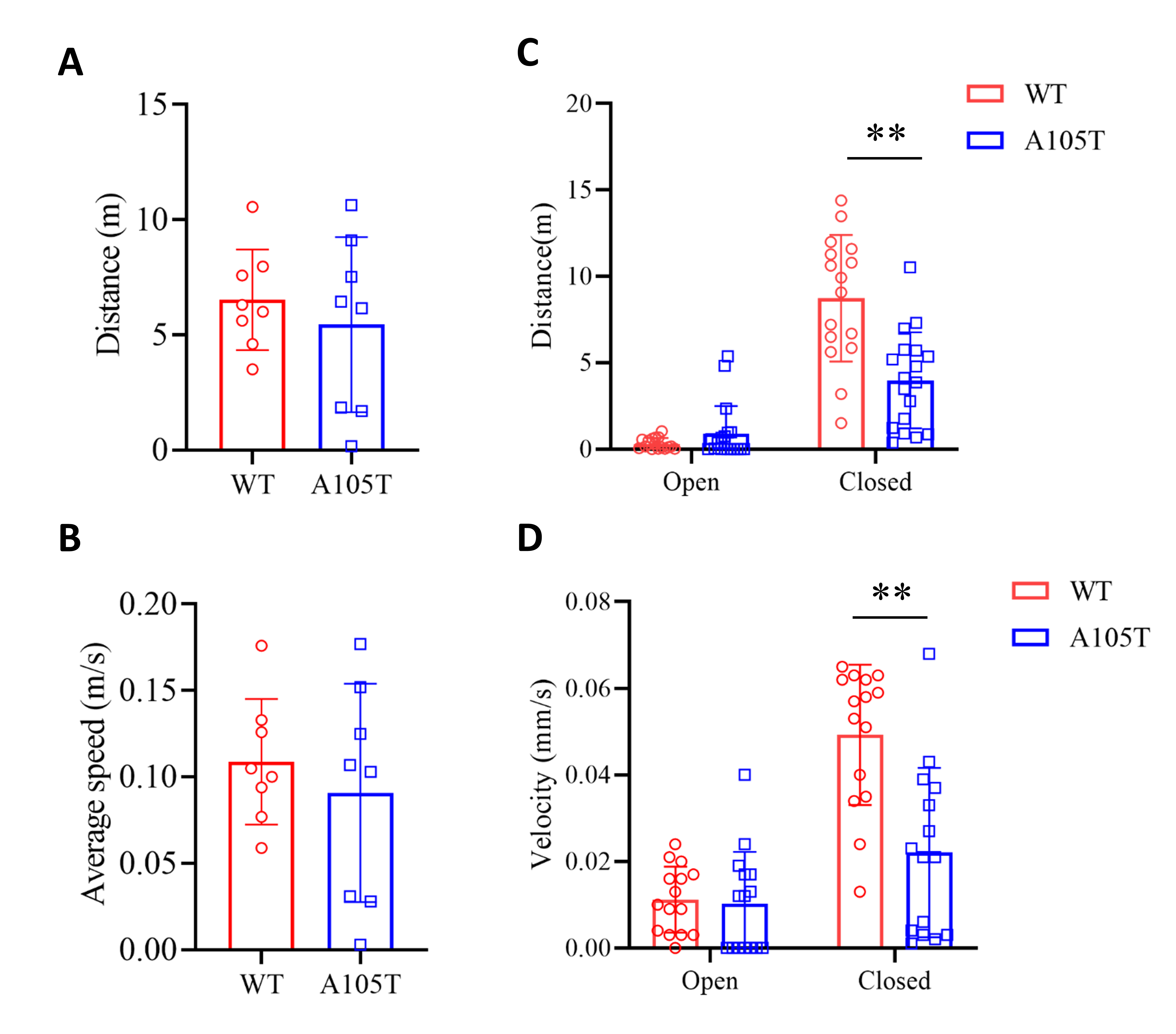

Supplement: Supplementary file 5 — Supplementary Figure 2 [file 41420_2025_2759_MOESM5_ESM.tif]

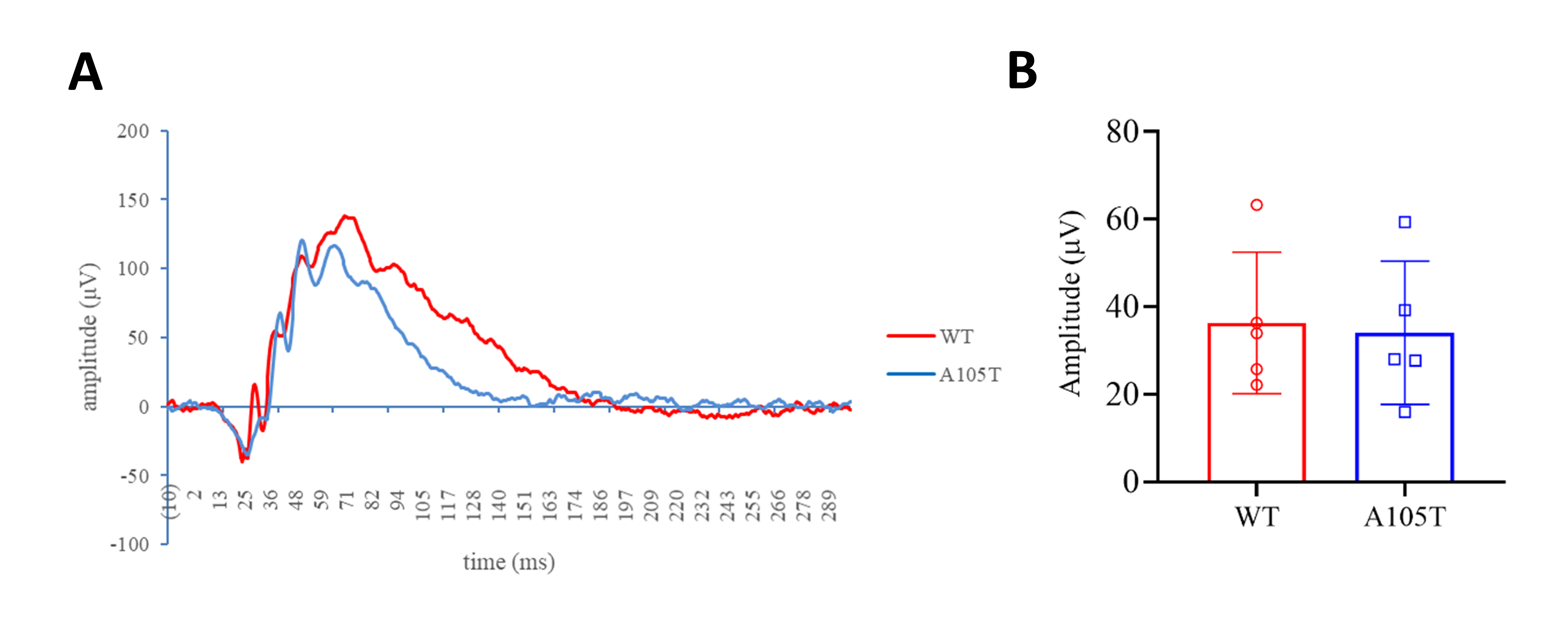

Supplement: Supplementary file 6 — Supplementary Figure 3 [file 41420_2025_2759_MOESM6_ESM.tif]

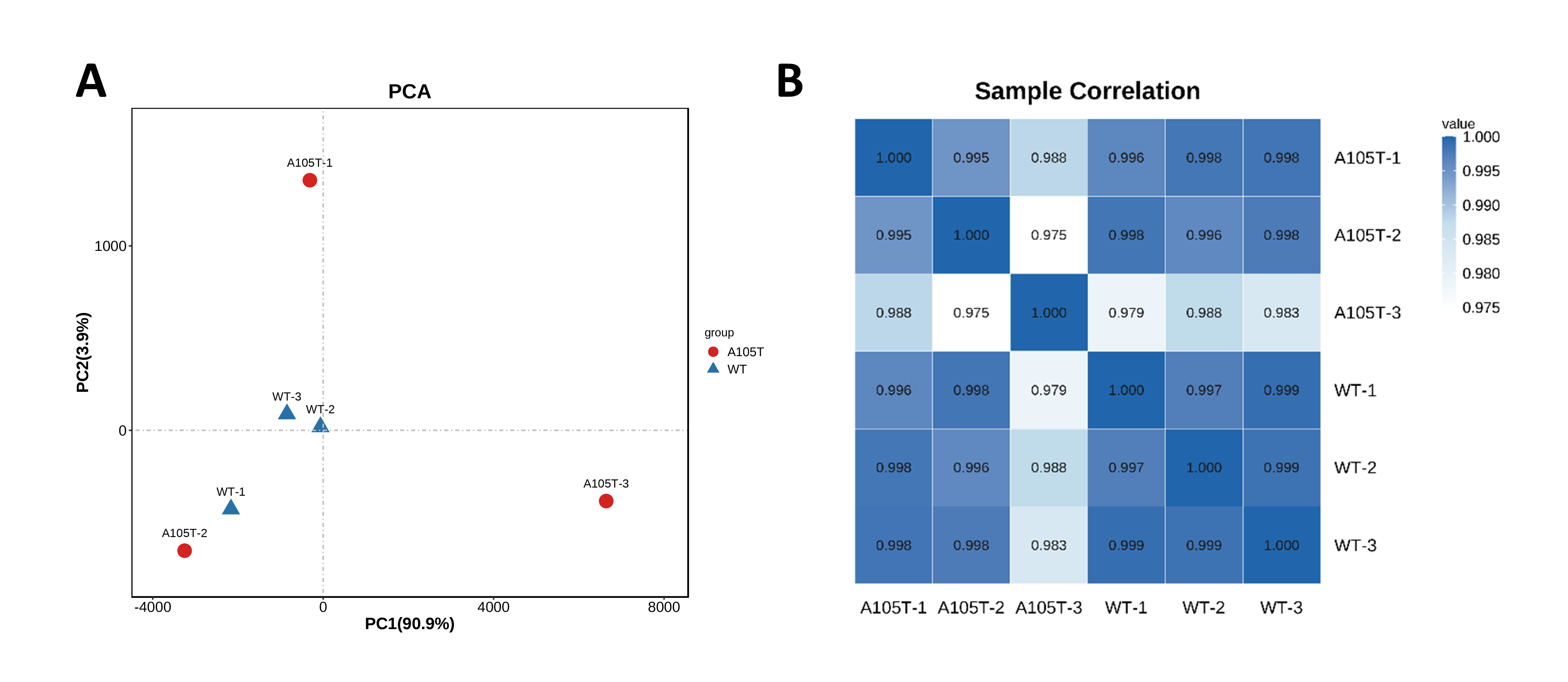

Supplement: Supplementary file 7 — Supplementary Figure 4 [file 41420_2025_2759_MOESM7_ESM.tif]

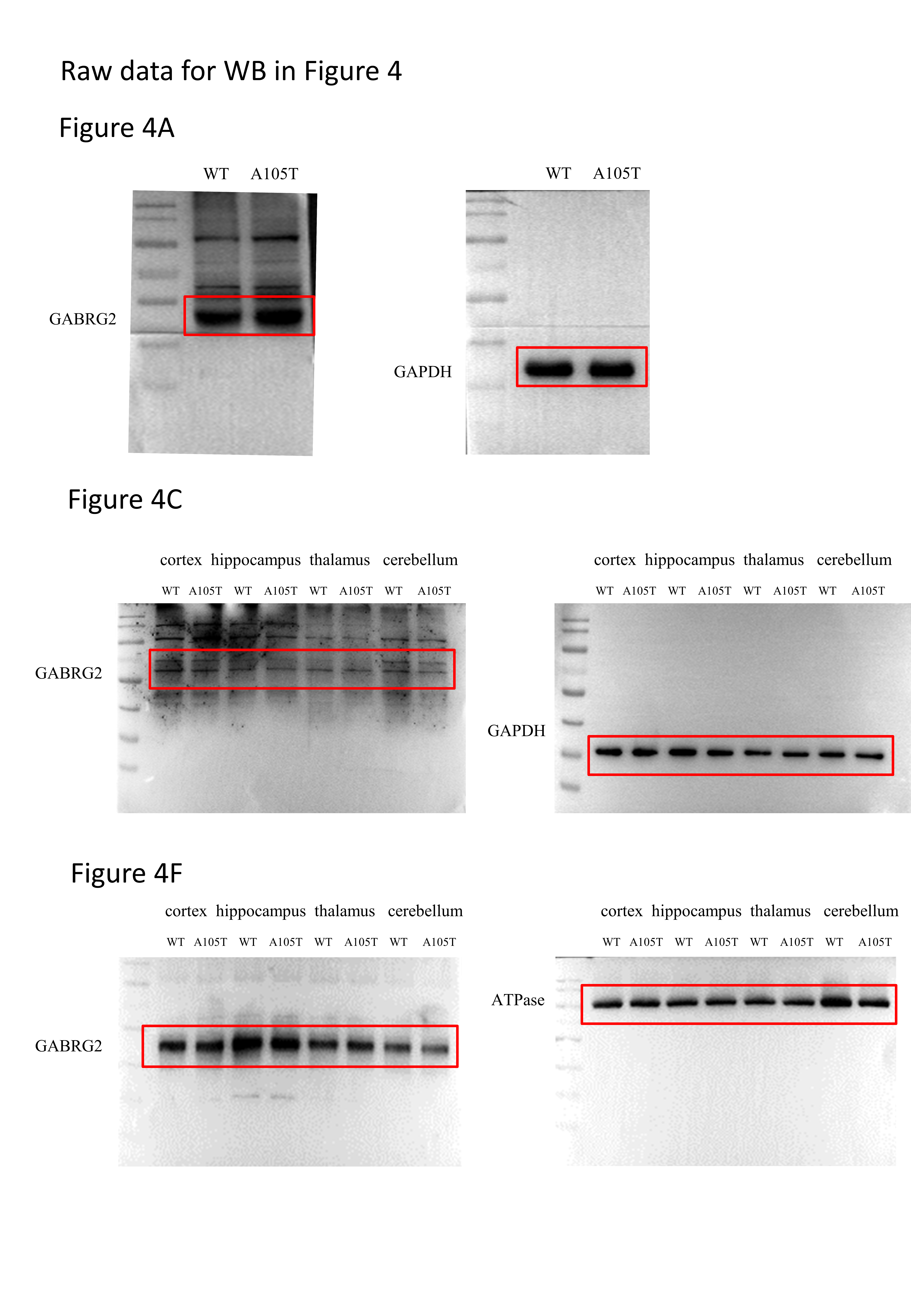

Supplement: Supplementary file 8 — original western blots [file 41420_2025_2759_MOESM8_ESM.tif]
